# ﻿*Euroscaptor darwini* sp. nov., a new species of mole (Mammalia, Eulipotyphla, Talpidae) from the north-central mountains in Vietnam

**DOI:** 10.3897/zookeys.1255.161942

**Published:** 2025-10-10

**Authors:** Son Truong Nguyen, Hai Tuan Bui, Vinh Quang Dau, Phuong Dinh Le, Yen Huong Vu

**Affiliations:** 1 Institute of Biology, Vietnam Academy of Science and Technology (VAST), 18 Hoang Quoc Viet Road, Hanoi, Vietnam Institute of Biology, Vietnam Academy of Science and Technology (VAST) Hanoi Vietnam; 2 Graduate University of Science and Technology, Vietnam Academy of Science and Technology (VAST), 18 Hoang Quoc Viet Road, Hanoi, Vietnam Graduate University of Science and Technology Hanoi Vietnam; 3 Faculty of Biology, Hanoi University of Science, Vietnam National University (VNU), 334 Nguyen Trai Road, Hanoi, Vietnam Hanoi University of Science Hanoi Vietnam; 4 Hong Duc University, 565 Quang Trung Road, Thanh Hoa, Vietnam Hong Duc University Thanh Hoa Vietnam; 5 Pu Luong Nature Reserve, Thanh Hoa, Vietnam Pu Luong Nature Reserve Thanh Hoa Vietnam

**Keywords:** Annamite Range, Darwin, mitochondrial gene, PCA, small mammal, taxonomy

## Abstract

A new species of fossorial mole (Eulipotyphla, Talpidae, *Euroscaptor*) is described from Pu Luong Nature Reserve, north-central Vietnam, based on distinct genetic and morphological characteristics. The species inhabits a geographically small and isolated upland patch (900–1100 m a.s.l.), sharply bounded by a nearly vertical escarpment. The new taxon is diagnosed by an extremely reduced tail both externally and osteologically, comprising only six or seven caudal vertebrae, the lowest number documented in the genus to date. The species differs further from known congeners in Southeast Asia by its slender cranium, narrow rostrum, elongated inner zygomatic arches, and significantly smaller anterior dentition. Phylogenetic analyses of the mitochondrial Cyt *b* gene indicate genetic distances of 5.41–6.35% from its closest relative, *E. subanura*, and clarify the evolutionary placement of the species within the genus. Multivariate analyses of 36 craniodental measurements identified key variables contributing to interspecific differentiation among Vietnamese moles, including breadth between infraorbital foramina, length of zygomatic arch, upper incisor–canine length, premolars length, and lower incisor–canine length. Specimens from the type locality show that females are larger than males. The discovery of this new *Euroscaptor* species currently raises the total number of recognized species in the genus to eleven worldwide and brings the number of fossorial mole species recorded in Vietnam to six. It highlights both the underestimated mammalian diversity of Vietnam and the importance of continued integrative surveys in montane landscapes, where micro-endemic and evolutionarily distinct taxa remain insufficiently documented and vulnerable to environmental change.

## ﻿Introduction

Vietnam, situated on the eastern boundary of the Indochinese peninsula and forming part of the Indo–Burma biodiversity hotspot, is recognized as one of the most biodiverse countries in Southeast Asia. This biodiversity is principally attributed to the country’s highly varied topography, complex climatic regimes, and biogeographic position within the transitional zone between the subtropics and tropics (Sterling and Hurley 2005; Sterling et al. 2006; Dang et al. 2008; Tordoff et al. 2012). Stretching more than 1650 km from north to south in a characteristic “S-shaped” configuration, Vietnam encompasses a mosaic of ecological regions, including river deltas, karst limestone formations, tropical lowland forests, and isolated high-elevation mountain ranges, each harboring distinct faunal assemblage (Bain and Hurley 2011). This habitat complexity, together with historical geological activity and limited faunal dispersal across rugged terrain, has led to significant biogeographic fragmentation and elevated levels of endemism terrestrial vertebrates (Sterling and Hurley 2005; Badgley et al. 2017).

Among small insectivorous mammals, fossorial mole species are especially sensitive to geographical barriers due to their highly specialized subterranean lifestyle and low dispersal capacity (Nevo 1985, 1999). These characteristics promote population isolation and localized divergence, making these insectivores an excellent model system for studying the effects of geographic isolation in driving genetic, morphological differentiation and speciation (Huxley 1938; Kottler 1978; Barrow and MacLeod 2008; Pérez et al. 2017). In Vietnam, the role of ecological gradients, especially altitudinal zonation, has been proposed as a major driver of both intraspecific and interspecific diversification in mammals (Motokawa and Lin 2002; Asahara et al. 2012; Bui et al. 2020a). The eastern mountain systems of Vietnam, particularly the Hoang Lien Son Range and Annamite Range, represent ancient montane landscapes that not only provide climatic and elevational refugia but also function as long-standing biogeographical barriers. These areas are highly conducive to allopatric speciation in limited dispersing taxa. In particular, montane habitats above 1000 meters have consistently yielded genetically and morphologically distinct populations, many of which have been overlooked in traditional faunal surveys due to their cryptic behaviors (Zemlemerova et al. 2016; Bui et al. 2020a).

The genus *Euroscaptor* Miller, 1940 (Talpini, Talpidae) is a taxonomically complex lineage of fossorial mammals, characterized by cryptic species and a broad distribution across East and Southeast Asia (Hutterer 2005; He et al. 2014; Kryštufek and Motokawa 2018; Burgin et al. 2020). Members of this genus are highly adapted to a subterranean lifestyle and typically inhabit forested mountain environments and moist montane habitats, across a wide elevational range. Despite their broad distribution, *Euroscaptor* species remain among the least known small mammals in Asia, having been historically under-sampled due to their elusive behaviors, cryptic habits, limited surface activity and morphologically conservative adaptations to underground niches.

*Euroscaptor* was originally established to accommodate a group of Asian moles exhibiting primitive dental characters and specialized fossorial morphologies (Kawada 2016). At present, it is recognized as the second largest genus of the family Talpidae (Kryštufek and Motokawa 2018; Burgin et al. 2020), comprising ten extant species, including: *E. micrura* (Hodgson, 1841), *E. longirostris* (Milne-Edwards, 1870), *E. klossi* (Thomas, 1929), *E. grandis* Miller, 1940, *E. parvidens* Miller, 1940, *E. malayana* (Chasen, 1940) (see Kawada et al. 2008), *E. subanura*Kawada et al., 2012, *E. kuznetsovi*Zemlemerova et al., 2016, *E. orlovi*Zemlemerova et al., 2016, and *E. ngoclinhensis*Zemlemerova et al., 2016 (see Bui et al. 2020a). Based on cranial morphology and dental characters, *Euroscaptor* species have been classified into two major clades: a western group and an eastern group (Kawada et al. 2009, 2012). The western group comprises *E. micrura*, *E. klossi*, and *E. malayana*, while the eastern group includes northern Vietnamese populations previously assigned to *E. longirostris*, which have now been reclassified into two separate species: *E. kuznetsovi* and *E. orlovi* (Zemlemerova et al. 2016), along with *E. parvidens*, *E. ngoclinhensis*, and *E. subanura*. Vietnam represents the highest diversity for the genus, currently harboring five recognized *Euroscaptor* species: *E. kuznetsovi*, *E. orlovi*, *E. parvidens*, *E. ngoclinhensis*, and *E. subanura*. Four of these species: *E. parvidens*, *E. ngoclinhensis*, *E. kuznetsovi* and *E. subanura*, are only restricted to Vietnam.

Phylogenetically, *Euroscaptor* forms a distinct clade within Talpinae, separate from other talpine genera such as *Mogera*, *Parascaptor*, and *Talpa* (Zemlemerova et al. 2013, 2016; Bannikova et al. 2015a). Recent advances in molecular systematics have revealed that *Euroscaptor* encompasses considerable cryptic diversity (He et al. 2014). Multilocus phylogenies based on mitochondrial genes (Cyt *b*, 12S rRNA) and nuclear loci (BRCA1, RAG1, ApoB) have revealed the several morphologically similar but genetically distinct lineages exist in this genus. These lineages often correspond to discrete geographic regions or elevational zones, suggesting allopatric speciation driven by mountainous topography and ecological barriers (Bannikova et al. 2015a, 2015b; Shinohara et al. 2015; Zemlemerova et al. 2016).

Morphologically, *Euroscaptor* species are characterized by external features associated with their underground-living lifestyle: a cylindrical body, robust forelimbs for digging, shovel-like claws, greatly reduced or absent external pinnae, minute and concealed eyes. Their rostrum is typically elongated and narrow, often with prominent sensory vibrissae (Kawada et al. 2012; Bui et al. 2020a). While external morphological characters are often conservative and overlapping among species, craniodental traits have proven to be more informative for species delimitation, when analyzed in combination with cytogenetic and molecular data (Kawada et al. 2012; Shinohara et al. 2015; Bui et al. 2020a). The skulls of *Euroscaptor* spp. are typically flat and elongated, with slender rostra, narrow interorbital widths, and highly variable palatal and braincase breadths. The genus retains a primitive dental formula: I3/3, C1/1, P4/4, M3/3 = 44 (Kawada et al. 2012). Interspecific distinctions often rely on subtle diagnostic features, such as the shape and alignment of molar cusps, the degree of triangularization of the upper molars, the presence or absence of the hypoconulid on lower molars, and the configuration of the zygomatic arches.

In the present study, we found five new specimens of *Euroscaptor* from montane forests above 1000 m in Pu Luong NR, north-central Vietnam. The new taxon is defined by a remarkably short tail, which is the most reduced among known congeners both in absolute length and in vertebral segmentation. Phylogenetic analyses based on Cyt *b* and 12S rRNA genes confirmed its distinct lineage, with genetic divergences falling within the expected range for interspecific differences in Talpidae. Multivariate analyses and morphometric comparisons of Vietnamese *Euroscaptor* populations were also conducted to assess morphological variation and further corroborate their taxonomic identity. Herein, we describe this newly discovered population as a new *Euroscaptor* species.

## ﻿Materials and methods

### ﻿Study locality and sampling methods

**Figure 1.**
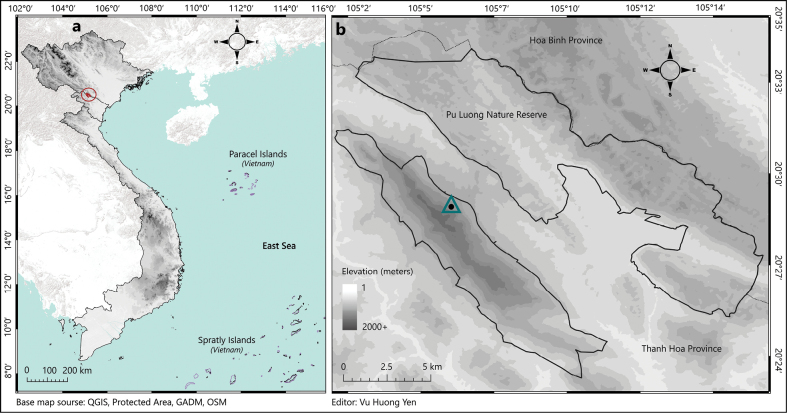
a. Map of Vietnam showing the location of Pu Luong NR in Thanh Hoa Province (highlighted in red); b. Black dot enclosed within teal triangle marks the type locality, distributed along altitudinal transects (600–1100 m a.s.l.). The map was generated using QGIS 3.38.3 (https://www.qgis.org) and edited in Adobe Photoshop 2023.

Pu Luong Nature Reserve (Pu Luong NR), situated in the northwestern region of Thanh Hoa Province, north-central Vietnam (20°21'–20°34'N, 105°02'–105°20'E) (see Fig. 1) (Le et al. 2015), is recognized as one of the key protected areas within the Indo–Burma Biodiversity Hotspot (BirdLife International Vietnam Programme and FIPI 2001; Tordoff et al. 2004). The reserve is characterized by a distinctive landscape formed by two parallel mountain ridges running northwest to southeast, separated by a central lowland valley, which is excluded from the core protected zone. The northeastern ridge consists primarily of highly dissected limestone karst formations, forming a geologic continuum with the karst system of Cuc Phuong NP and extending further northwest ward to Son La Province, whereas the southwestern ridge is characterized predominantly by typical non-karstic (soil-based) mountain terrain. Elevations within Pu Luong range from ca 60 m to more than 1700 m a.s.l., harboring diverse microhabitats from lowland tropical broadleaf forests to montane evergreen ecosystems (Center for Nature Conservation and Development 2005).

Our field surveys targeting small mammals, particularly fossorial moles, were conducted along the trail to the summit of Pu Luong Mountain, with the assistance of a local guide across two periods: the first from 5–12 November 2024, and the second from 4–10 April 2025. Sampling was concentrated along the southwestern ridge of Pu Luong Mountain, with survey effort directed toward forest habitats between 900 m and 1100 m a.s.l., during the dry season. Sampling was conducted using specialized tunnel traps, a standard method for capturing fossorial mammals (Sikes et al. 2011; Kawada et al. 2012). Each tunnel trap consisted of a cylindrical plastic tube (~4.5 cm in diameter and ~8.5 cm in length), with a central chamber fitted with a spring-loaded mechanism. Traps were checked daily early morning to ensure proper functioning and to promptly retrieve captured specimens.

**Figure 2.**
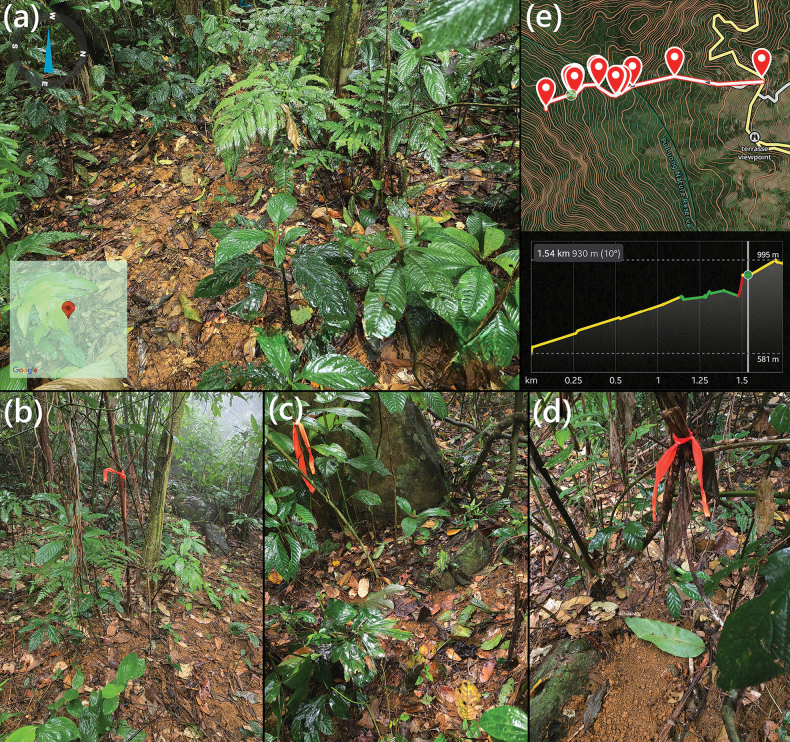
Habitat characteristics and sampling localities in Pu Luong NR. a. Tropical evergreen forest at ~935 m a.s.l., representing the type locality of the holotype; b–d. Typical microhabitats where paratypes were captured. Sampling points (red markers) correspond to trapping stations along the altitudinal transect; e. Topographic trail map and elevational profile (581–995 m a.s.l.) of the sampling route, generated using the Gaia GPS mobile application.

During each survey, eleven tunnel traps were deployed at six trapping stations along forest trails and areas with fresh molehills, targeting locations with soft, moist soils indicative of recent mole activity. The stations were established at ~950 m a.s.l. in a relatively flat area beyond an almost vertical slope (Figs 1, 2a), which corresponding to the site where the first unknown mole specimen, an adult male, was discovered in November 2024 (Fig. 1). During the follow-up field survey in April 2025, the research team collected two additional individuals from the same locality where the first male had been obtained, and two others from the elevation of ca1000 m a.s.l. (Fig. 2b–d). All specimens were captured using tunnel traps placed along altitudinal range (950–1100 m) across various habitat types. Geographic coordinates and elevations were recorded using the Gaia GPS mobile application (WGS84 datum) (Fig. 2e).

Following processing, captured individuals were retained as voucher specimens for taxonomic identification and further examination. All voucher specimens were initially fixed in 95% ethanol for the first 30 days, and then transferred to 70% ethanol for long-term storage. The skull, pelvic bone, and caudal vertebrae were extracted from the specimen and thoroughly cleaned. Liver tissue samples were separately preserved in absolute ethanol for subsequent genetic analysis. Voucher specimens and associated tissue samples of new taxon are currently deposited in the collections of the Department of Zoology, Institute of Biology (**IB**), VAST, Hanoi.

### ﻿Molecular data and phylogenetic analyses

DNA was isolated from liver tissue samples preserved in 99% ethanol using the DNeasy® Blood and Tissue Kit (Qiagen, Hilden, Germany), following the manufacturer’s standard protocol. The mitochondrial Cyt *b* gene was selected due to its demonstrated effectiveness in resolving species-level identification. Amplification of the Cyt *b* fragment was performed using the primer pair SoriF (5′–CCATCTCTGGTTTACAAGAC–3′) and SoriR (5′ TGACATGAAAAATCATCGTTG–3′) (Bui et al. 2020b). In addition, the partial mitochondrial 12S ribosomal RNA (12S rRNA) gene was included in the analysis to provide complementary phylogenetic resolution. This fragment was amplified using the primer pair L–613 (5′–ACACAAAGCATGGCACTGAA–3′; Mindell et al. 1991) and H–1478 (5′–TGACTGCAGAGGGTGACGGGCGGTGTGT–3′; Kocher et al. 1989). PCR reactions were conducted using the PCR Master Mix (Phusa Genomics, Can Tho, Vietnam) under the following thermal cycling conditions: an initial denaturation at 95 °C for 5 min; followed by 35 cycles of 95 °C for 30 s, annealing at 56 °C for 50 s, and extension at 72 °C for 2 min; with a final extension at 72 °C for 10 min. Amplified products were then purified and sequenced by 1^st^ BASE (Selangor, Malaysia) using Sanger sequencing protocols.

Forward and reverse chromatograms of both Cyt *b* and 12S rRNA sequences were manually edited and assembled into consensus sequences using Chromas Pro (Technelysium Pty Ltd., Australia), MEGA 11 (Tamura et al. 2021) and BioEdit (Hall 1999), with manual verification for base-calling accuracy and alignment consistency. Multiple sequence alignment was performed using the MUSCLE algorithm (Edgar 2004) implemented in MEGA 11. The final alignment lengths were 1140 base pairs for Cyt *b* and 846 base pairs for 12S rRNA. No insertions or deletions (indels) were detected across any of the ingroup sequences. The concatenated alignment of Cyt *b* and 12S rRNA gene fragments was constructed using SequenceMatrix v. 1.8 (Vaidya et al. 2011), based on aligned sequences with matching taxon labels. Intraspecific and interspecific pairwise uncorrected divergences (*p*–distances) for Cyt *b* sequences were computed in MEGA 11, applying pairwise deletion of gaps/missing sites and variance estimates via bootstrap with 10000 replicates.

Phylogenetic reconstruction was inferred using both Maximum Likelihood (ML) and Bayesian Inference (BI) methods. The ML analysis was conducted using IQ–TREE v. 1.6.12 (Nguyen et al. 2015), with the optimal substitution model for each dataset determined by the Bayesian Information Criterion (BIC) via ModelFinder (Kalyaanamoorthy et al. 2017), as implemented in IQ–TREE. Model selection identified HKY+I as the best-fit model for the Cyt *b*, GTR+G+I for the 12S rRNA, and TN93+G for the concatenated alignment. ML trees were generated with 10000 ultrafast bootstrap replicates (UFBoot, Hoang et al. 2018) to evaluate branch support. Bayesian phylogenetic inference was performed using MrBayes v. 3.2.7 (Ronquist et al. 2012). Substitution models for each partition were selected using Kakusan4 (Tanabe 2011) under Akaike Information Criterion (AIC). Each analysis consisted of two independent runs of four Markov Chain Monte Carlo (MCMC) chains each (one cold and three heated) for 10 million generations, sampling every 1000 generations. A 50% majority-rule consensus tree was constructed after discarding the first 25% of trees as burn-in, and Bayesian posterior probabilities (PP) were calculated for all nodes. The resulting phylogenetic trees were visualized using FigTree v. 1.4.4 and represented graphically in Adobe Illustrator 2023.

### ﻿Morphological examination

All five captured individuals were photographed after capture using a Canon EOS Kiss X7 digital camera equipped with a Canon EF-S 18–55 mm f/3.5–5.6 kit lens. Standard morphometric measurements were recorded, followed procedures in Kawada et al. (2012). The following measurements were obtained: body length (**HB**), tail length (**T**), hind foot length with claws (**HF**), forefoot width (**FFW**), and forefoot length without claws (**FFL**), along with data on sex, and reproductive status.

**Figure 3.**
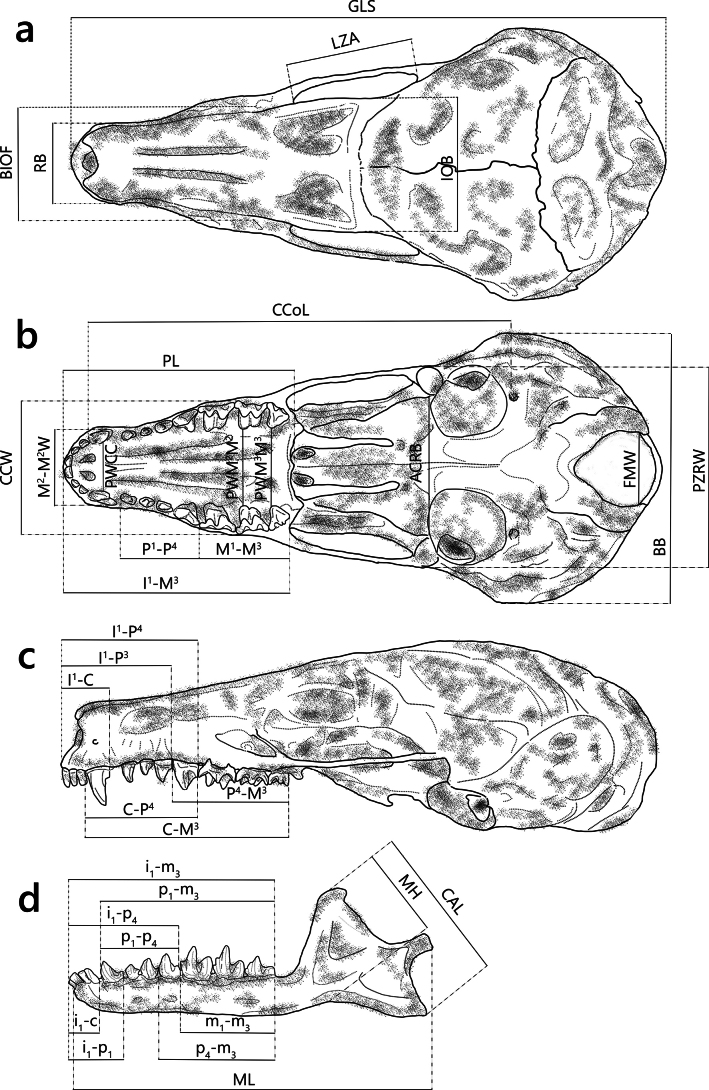
Dorsal (a), ventral (b), and lateral (c) views of the cranium; d. Lateral view of the mandible showing craniodental measurements. Diagram constructed based on the craniodental morphology of the new male specimen by VHY.

Craniodental measurements were taken under a stereoscopic microscope (SMZ 745, Nikon, Japan) using an electronic digital caliper (NTD12–15PMX, Mitutoyo, Japan) with a precision of 0.01 mm. A total of 36 craniodental measurements were taken for each specimen, including 15 metrics following the method of Kawada et al. (2007), and an additional 21 measurements recorded in this study (Fig. 3). Detailed descriptions of each measurement are provided in Suppl. material 1. The dataset comprised measurements from 65 adult skulls, including five skulls of new *Euroscaptor* and 60 comparative skulls from other *Euroscaptor* species recognized in Vietnam (Suppl. material 2). All the measurements are in millimeters.

### ﻿Statistical analyses

Minimum, maximum, mean values, and standard deviations for measurements were calculated using Microsoft^®^ Excel version Office 2021 (Microsoft, Redmond, WA, USA). Multivariate analyses of craniodental morphology among Vietnamese fossorial moles were conducted to evaluate interspecific morphometric variations, including Principal Component Analysis (PCA) and Multivariate Analysis of Variance (MANOVA) based on log-transformed measurements. Differences in the mean values between groups were examined using t-tests, preceded by F-tests to assess homogeneity of variances (P < 0.05). Analysis of variance (One-way ANOVA) followed by Tukey’s honestly significant difference (HSD) (significant at P < 0.05) was used for pairwise comparisons within taxa. All these analyses were performed using the PAST software v. 4.13 (Hammer et al. 2001).

## ﻿Results

### ﻿Phylogenetic analyses

**Figure 4.**
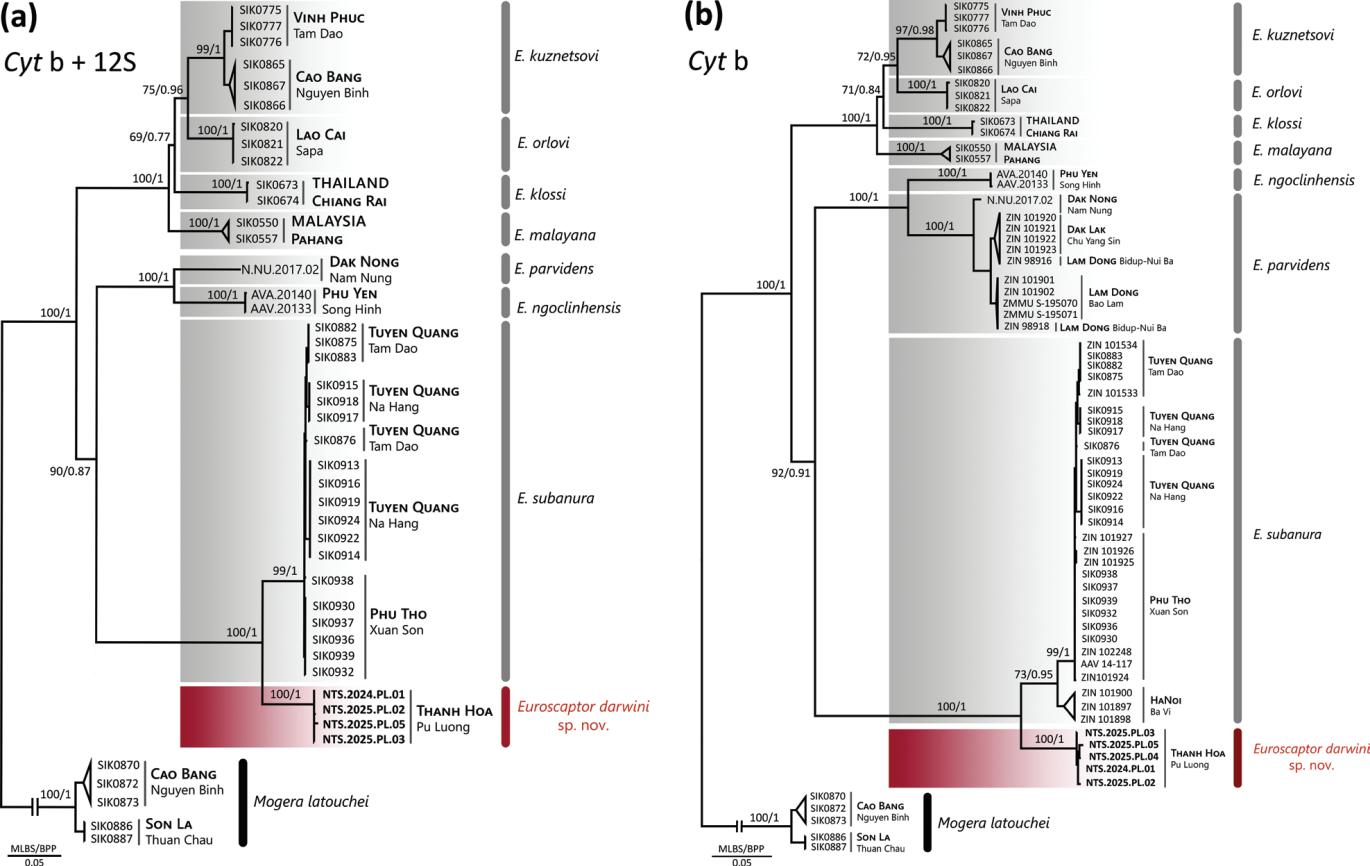
Maximum likelihood (ML) phylogenetic tree constructed based on a. The concatenated mitochondrial Cyt *b* and 12S rRNA gene sequences (1,986 bp); b. Cyt *b* sequences (1,140 bp) of the *Euroscaptor* spp. Nodal support values are indicated as Maximum Likelihood Bootstrap Support/Bayesian Posterior Probability (MLBS/BPP). Representatives of the genus *Mogera* were included as the outgroup. Each terminal taxon is labelled with its corresponding voucher number, sampling locality, and GenBank Accession Number of both Cyt *b* and 12S gene listed in Suppl. material 5.

The concatenated phylogenetic tree reconstructed using both ML and BI methods generated congruent topologies with strong statistical support across all major nodes. ML tree is presented in Fig. 4a, with support values indicated as ML bootstrap (MLBS) and Bayesian posterior probabilities (PP). The genus *Euroscaptor* was recovered as a well-supported monophyletic group, clearly distinguished from the outgroup *Mogera latouchei* in Vietnam. All recognized species-level clades exhibit high support values (MLBS ≥ 90%).

The newly collected specimens from Pu Luong NR represent a genetically distinct and maximally supported monophyletic lineage (MLBS = 100%, PP = 1.0). This clade is positioned as the sister group to the *E. subanura*, from which it is separated by strong branch support and considerable genetic distance. Besides, the new collected mole groups with *E. subanura*, forming a well-supported monophyletic clade (100/1), separate it from other Vietnamese congeners, including *E. parvidens* and *E. ngoclinhensis*. This phylogenetic placement presents that new collected individuals and *E. subanura* share a more recent common ancestor with each other than with any other moles in Vietnam. Intraspecific genetic divergence within this population is minimal (Cyt *b*: 0.09–0.35%). The pairwise genetic distances based on Cyt *b* (Table 1) indicate the distinctiveness of newly discovered *Euroscaptor* species. The uncorrected *p*–distance between this population and the *E. subanura* population ranges from 5.41% to 6.35%, which is within the generally accepted threshold for species-level divergence in mammals (typically 2–11%) (Bradley and Baker 2001; Baker and Bradley 2006). Genetic divergence among *E. subanura* populations is also evident (Table 2). Among four populations of *E. subanura* distributed in Vietnam, the lowest interspecific distance (5.41%) was recorded between *E. darwini* and the *E. subanura* population from Ba Vi NP, while the highest (6.35%) was observed with the Na Hang population. Particularly, the Ba Vi population appears genetically distinct from other *E. subanura* populations, forming a weakly supported lineage (MLBS = 73%, BPP = 0.95) (Fig. 4b). Despite being morphologically similar, this population shows pairwise divergences of 1.93–2.54% with other *E. subanura* populations.

**Table 1.** Uncorrected pairwise distances matrix (*p*–distance) showing genetic divergences (%, minimum–maximum) for the Cyt *b* gene among *Euroscaptor* species included in this study. The list of Cyt *b* sequences used for pairwise comparisons is provided in Suppl. material 3.

Species		n	1	2	3	4	5	6	7	8
1	*E. darwini* sp. nov.	5	0.09–0.35							
2	*E. subanura*	30	5.44–6.35	0.09–2.45						
3	*E. ngoclinhensis*	2	14.94–15.38	13.97–14.50	0.18					
4	*E. parvidens*	11	14.95–15.88	14.65–15.44	8.17–8.70	0.09–2.11				
5	*E. kuznetsovi*	6	14.82–15.44	14.29–15.44	12.30–13.18	11.07–12.54	0.00–1.49			
6	*E. orlovi*	3	15.79–15.96	15.18–16.32	12.30–12.65	12.28–12.81	5.61–5.87	0.00–0.18		
7	*E. klossi*	2	15.88–15.96	15.26–16.14	13.62–13.80	12.72–13.07	7.72–8.51	7.82–7.98	0.18	
8	*E. malayana*	2	14.56–14.82	14.30–15.18	12.74–13.36	12.13–13.60	6.58–7.45	7.28–7.81	8.42–8.60	0.88

**Table 2.** Uncorrected pairwise distances matrix (*p*–distance) showing genetic divergences (%, minimum–maximum) for the Cyt *b* gene between the newly described *Euroscaptor* species and four *E. subanura* populations from Vietnam included in phylogenetic analyses. The raw *p*–distance matrix of Cyt *b* sequences used for comparisons is provided in Suppl. material 4.

*n*	Species	*E. darwini* sp. nov. (Pu Luong NR pop.)	*E. subanura* (Tam Dao pop.)	*E. subanura* (Ba Vi NP pop.)	*E. subanura* (Xuan Son NP pop.)	*E. subanura* (Na Hang pop.)
5	*E. darwini* sp. nov. (Pu Luong NR pop.)	0.09–0.35				
6	*E. subanura* (Tam Dao pop.)	5.53–5.96	0.00–0.35			
3	*E. subanura* (Ba Vi NP pop.)	5.41–5.61	2.19–2.46	0.09–1.32		
12	*E. subanura* (Xuan Son NP pop.)	5.44–5.71	0.18–0.44	1.93–2.20	0.00–0.18	
9	*E. subanura* (Na Hang pop.)	5.70–6.35	0.35–0.61	2.19–2.54	0.26–0.53	0.00–0.61

Comparisons between new *Euroscaptor* species with other congeners reveal substantially higher levels of divergence: reaching 14.94–15.38% with *E. ngoclinhensis*, 14.95–15.88% with *E. parvidens*, 14.82–15.44% with *E. kuznetsovi*, and up to 15.96% with *E. klossi*. The phylogeny resolves two major evolutionary lineages within *Euroscaptor*, consistent with previous studies (Shinohara et al. 2014; Zemlemerova et al. 2016). The first lineage comprises a widely distributed clade (*E. kuznetsovi*, *E. orlovi*, *E. klossi*, and *E. malayana*), occurring across southern China, northern Vietnam, Thailand, and Malaysia. The second represents of a southwestern Indochinese clade, including *E. subanura*, *E. parvidens*, *E. ngoclinhensis*, and the new *Euroscaptor* species from north-central Vietnam).

Based on genetic comparisons between the newly collected specimens from the Pu Luong population and other *Euroscaptor* species, combined with the morphological differences presented below, we describe a new species: *Euroscaptor darwini* sp. nov.

### ﻿Taxonomic account

**Mammalia Linnaeus, 1758**

**Eulipotyphla Waddell et al., 1999**

**Talpidae G. Fischer, 1814**

***Euroscaptor* Miller, 1940**

#### ﻿ Euroscaptor darwini sp. nov.

Taxon classificationAnimaliaEulipotyphlaTalpidae

4E787F99-92EF-55A8-8E49-17C3F85D68BC

https://zoobank.org/A8092515-D296-4C0D-ADA9-82A8E0ACF144

Figs 1, 2, 3, 4, 5, 6, 7, 8, 9, Tables 3, 4, 5, 6, 7

##### Material examined.

***Holotype***: Vietnam • ♂; Thanh Hoa Province, Ba Thuoc District, Thanh Son Commune, Pu Luong Nature Reserve; 20°29.01'N, 105°6.11'E; approx. 935 m a.s.l.; 10 Nov. 2024; N. V. Ngan leg.; NTS.2024.PL.01; IB–VAST. ***Paratypes***: Vietnam • 2 ♀♀; same locality as for holotype; same coordinates; same altitude; 9 Apr. 2025; S. T. Nguyen, Y. H. Vu and N. V. Ngan leg.; NTS.2025.PL.02, NTS.2025.PL.03; IB–VAST; and 2 ♀♀; same locality as for holotype; 20°28.95'N, 105°6.02'E; approx. 1007 m a.s.l.; 13 Apr. 2025; N. V. Ngan leg.; NTS.2025.PL.04, NTS.2025.PL.05; IB–VAST.

##### Diagnosis.

*Euroscaptor darwini* sp. nov. is clearly distinguished from congeners by its extremely short, vestigial tail, which protrudes slightly less than 2 mm beyond the skin surface. It is entirely covered by short, sparse bristle hairs that progressively lengthen toward the distal end, reaching approximately twice the length of the underlying tail. The tail is composed of only six or seven caudal vertebrae, significantly fewer than in other *Euroscaptor* species. The interorbital region is moderately narrow, with the inter-foraminal distance between the infraorbital foramina being conspicuously constricted. The zygomatic arches are weakly developed but exhibit an atypically elongated form. The osseous junction between the infraorbital foramina and the palate is slender and lacks lateral expansion. In lingual view of mandible, the fourth lower premolar and all three lower molars have crowns that are broader than height, with overall small tooth dimensions. The mandible is delicate, characterized by a narrow ascending ramus and fragile angular process. The pelvic girdle is delicate, markedly reduced in both size and structural robustness.

##### Description of the holotype.

***External morphology*.** The holotype of *Euroscaptor darwini* sp. nov. (NTS.2024.PL.021) is an adult male with a compact body and small overall size (HB = 115.71 mm) (Table 3). The body is stream-lined and the head is conical, tapering smoothly anteriorly toward the snout. The body appears muscular, with a thick neck and prominent scapular region. The thorax and abdomen are proportionally broad, and the trunk is laterally compressed in dorsal view. The snout is elongated, naked, and prominently pink, with the distal portion slightly expanded into a bulbous structure. Numerous minute wart-like protuberances and well-developed vibrissae are present on the lateral and dorsal surface of the rhinarium for highly tactile function. The eyes are vestigial and not externally visible, deeply recessed beneath the skin. External pinnae are entirely absent. The nostrils open anteriorly and slightly laterally. The forelimbs are robust, with large palms and hyperdeveloped claws. Digits III and IV possess broad, chisel-shaped claws, while digit I bears a shorter but similarly broadened claw. The hindlimbs are more gracile but remain sturdy. The palms and soles are sparsely covered in naked skin and bear slightly keratinized pads.

**Table 3.** Comparative statistics of external measurements (in millimeters) between *E. darwini* sp. nov. and other fossorial mole species. (*) indicates shared measurements between *E. parvidens* and *E. ngoclinhensis*; (#) indicates shared measurements between *E. kuznetsovi* and *E. orlovi*.

Species	Reference (*n*)	HB	T	HF	FFW	FFL	Tail ratio = T/HB (%)
*E. darwini* sp. nov.	This study (holotype ♂)	115.71	2.11	14.51	13.88	13.29	1.82
	This study (4 paratypes ♀)	122.21–127.93 125.52 ± 2.86	2.67–3.74 3.33 ± 0.47	14.86–15.38 15.03 ± 0.24	14.02–14.51 14.21 ± 0.22	13.64–14.11 13.86 ± 0.28	2.18–2.92 2.64 ± 0.32
	This study (both sexes)	115.71–127.93 123.56 ± 5.04	2.11–3.74 3.08 ± 0.68	14.51–15.38 14.93 ± 0.31	13.88–14.51 14.14 ± 0.24	13.29–14.11 13.79 ± 0.41	1.82–2.92 2.48 ± 0.46
*E. subanura*	Kawada et al. (2012) (8)	113.0–130.0 122.88 ± 5.51	4.0–5.0 4.38 ± 0.44	13.5–15.5 14 ± 0.38	15.0–16.5 14.38 ± 0.38	14.0–15.5 14.75 ± 0.46	3.6
	Kawada (2016) (28)	113.0–131.5 125.48 ± 3.95	2.5–5.0 3.80 ± 0.79	13.0–14.5 13.70 ± 0.49	13.0–15.5 14.21 ± 0.55	13.0–15.5 14.27 ± 0.62	1.96–4.24 3.04 ± 0.65
	Bui (2022) (42)	107.0–130.5 121.89 ± 5.61	2.50–5.00 3.76 ± 0.73	12.0–16.5 14.02 ± 0.87	13.0–15.5 14.29 ± 0.55	13.80–18.00 14.80 ± 0.84	2.33–3.85 3.08 ± 0.56
*E. parvidens*	Kawada et al. (2012) (10) *	135.0–148.5 143.75 ± 3.75	5.5–9.0 6.95 ± 1.09	13.5–15.5 14.6 ± 0.66	15.0–16.5 15.4 ± 0.61	14.5–16.5 15.3 ± 0.63	4.8
	Kawada (2016) (18) *	135.0–150.0 144.89 ± 3.77	5.5–10.5 7.42 ± 1.30	13.5–16.0 14.92 ± 0.69	14.0–17.5 15.47 ± 0.88	14.5–18.0 15.75 ± 0.93	4.07–7.07 5.11 ± 0.83
	Bui (2022) (42)	130–165.5 150.9 ± 10.71	5.5–10.0 7.9 ± 1.48	12.0–17.74 16.01 ± 1.18	14.15–16.96 15.65 ± 0.54	14.7–17.6 16.03 ± 0.80	4.23–6.06 5.23 ± 0.0.75
*E. ngoclinhensis*	Bui (2022) (30)	120.0–150.0 137.87 ± 9.01	5.50–10.0 7.50 ± 1.18	12.0–16.0 14.15 ± 0.93	12.27–17.0 14.66 ± 1.34	13.20–18.00 15.02 ± 1.09	4.58–6.67 5.44 ± 0.86
*E. kuznetsovi*	Kawada et al. (2012) (19) #	133.0–144.0 138.45 ± 3.39	14.0–17.0 15.37 ± 1.01	14.5–16.5 15.55 ± 0.50	14.5–17.0 15.95 ± 0.71	15.0–17.5 16.26 ± 0.77	11.1
	Kawada (2016) (27) #	122.0–144.0 136.85 ± 4.97	10.0–17.0 14.69 ± 2.00	14.5–16.5 15.46 ± 0.46	14.5–17.0 15.91 ± 0.72	15.0–17.5 16.24 ± 0.74	8.19–11.81 10.73 ± 0.93
	Zemlemerova et al. (2016)	132–136					11.4–12.5
	Kryštufek and Motokawa (2018)	117–136	14–17	14.5–16.5			11.6–14.5
	Bui (2022) (24)	115.4–144 137.37 ± 5.74	14–17.5 15.65 ± 1.09	14.5–18.4 15.73 ± 0.77	14.5–17 15.82 ± 0.69	15–17.9 16.36 ± 0.80	11.33–12.57 12.05 ± 0.4
*E. orlovi*	Zemlemerova et al. (2016)	115–129					12.6–13.7
	Kryštufek and Motokawa (2018)	106–129	15–17.5	15.5			12.5–15.1
	Bui (2022) (6)	115–137.62 127 ± 7.40	11.76–19.28 16 ± 2.29	13.43–15.5 14.87 ± 0.92	14–16.5 15.15 ± 0.73	15–16.5 15.83 ± 0.76	10.63–13.57 12.45 ± 0.67
*E. longirostris*	Smith and Xie (2008)	90–145	11.0–25	14–23			
*E. klossi*	Kawada et al. (2012) (2)	130.0–135.5	9.5–10.5	15	16	16.0–16.5	7.5
	Kawada (2016) (4)	115.0–135.5 127.63 ± 8.81	9.5–13 11.25 ± 1.55	15.0–16.0 15.38 ± 0.48	16.0–17.0 16.38 ± 0.48	15.5–16.5 16.13 ± 0.48	7.01–11.30 8.91 ± 1.84
	Smith and Xie (2008)	115–135.5	9.5–13	15–16			7–11.3
*E. malayana*	Kawada et al. (2008)	124–134.5	4.5–8.5	14–16.5	15–17	15–19	3.35–6.59
	Kawada (2016) (10)	128.5–134.5 131.15 ± 1.94	4.5–8.5 5.70 ± 1.09	14.5–16.5 15.50 ± 0.62	15.5–17.0 16.40 ± 0.57	15.0–16.5 15.80 ± 0.42	3.34–6.58 4.35 ± 0.86
	Smith and Xie (2008)	128.5–134.5	4.5–8.5	14.5–16.5			3.3–6.6
*E. grandis*	Miller (1940)	150	9.6	18	15		
*E. micrurus*	Smith and Xie (2008)	128–135	5–9	15–16			

***Pelage coloration*.** Dense, plush, and velvety, the dorsal pelage is a uniform dark greyish-black, with a faint silvery reflection on the hair tips creating a subtly iridescent, metallic sheen under direct light. The lateral flanks blend seamlessly into the ventral region. The ventral fur is slightly lighter, ranging from dusky grey to smoky brown, most prominent around the throat, chest, and inguinal regions. The fur around the muzzle, vibrissae base, and forefoot digits is short and uniform in color. The forefeet and hindfeet are sparsely furred and show exposed pinkish skin around the digits and palms. The tail is uniformly dark grey and bristle-covered.

**Figure 5.**
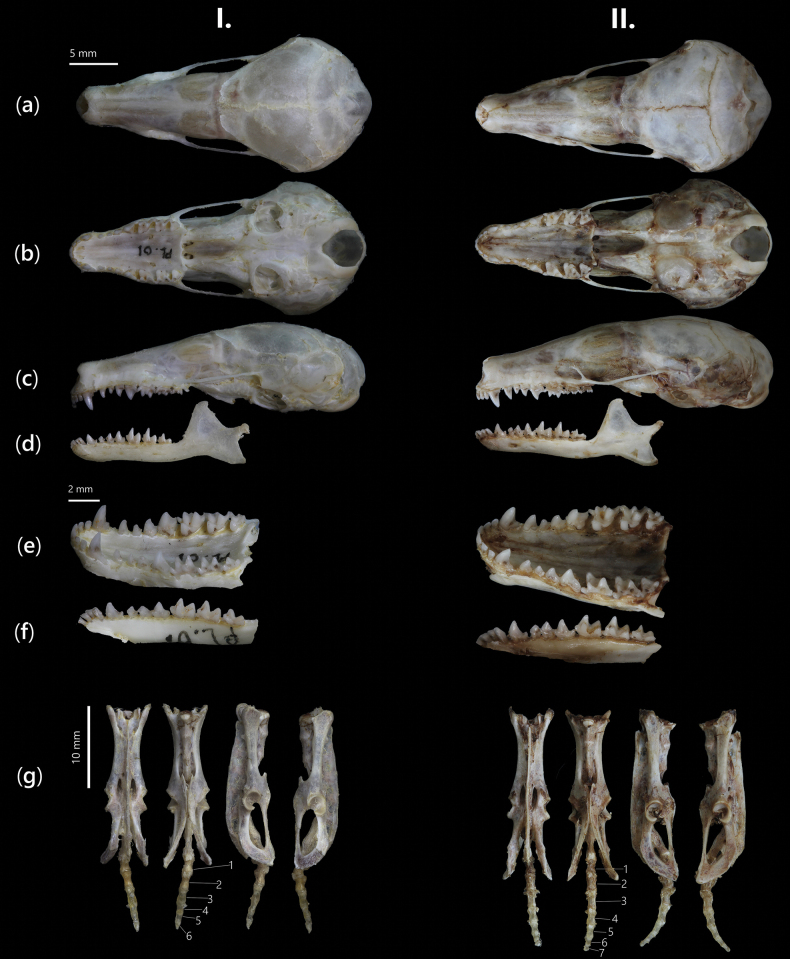
Cranium, mandible, and pelvic bone of *E. darwini* sp. nov.: (I) ♂ holotype, NTS.2024.PL.01, and (II) ♀ paratype, NTS.2025.PL.02. From top to bottom: a. Dorsal; b. Ventral; c, d. Lateral views of cranium and mandible, respectively; e, f. Lingual view (right side) of upper toothrow and lower toothrow, respectively; g. Four aspects of pelvis bone and caudal vertebrae.

***Tail and caudal vertebrae*.** The tail of *E. darwini* is extremely short, vestigial, with only approximately 1.5 mm protruding beyond the skin surface. The tail is nearly flush with the skin surface, with only a minute tip visible beyond the pelage. It is completely covered in sparse, short bristle-like hairs, forming a slightly tufted appearance. Osteological examination revealed a reduction in tail length, underlain by the presence of only 6 caudal vertebrae which is representing the most extreme case of caudal shortening documented within *Euroscaptor* (Fig. 5).

***Forefeet and claws*.** The forefeet are proportionally large and robust, with hypertrophied palms and prominent digital claws, particularly on digits III and IV. The first digit bears a shorter but stout claw. The claws are strongly recurved and lightly pigmented with ivory tips. Hindfeet are slender, lacking the specialization seen in the forefeet.

***Cranium morphology*.** The cranium is diminutive, lightly built, and slender in profile, with a greatest length (GLS) of 30.51 mm falling within the lower size range documented for *Euroscaptor* species in Vietnam (Table 4). The rostrum is proportionally short and gradually tapered anteriorly, forming a triangular outline in dorsal view. In lateral aspect, the rostral region is straight and only slightly arched dorsally. The braincase is moderately inflated with a smoothly domed dorsal surface. The interorbital region is nearly parallel-sided, maintaining consistent height from the frontals to the parietals. The sagittal crest is absent, and the lambdoidal crest is low and rounded, contributing to the smooth contour of the posterior cranial vault. The zygomatic arches are poorly developed, lacking the thickened and laterally expanded morphology seen in larger species (Fig. 5c). The infraorbital foramina are small and positioned close to the anterior margin of the maxillae. The auditory bullae are dorsoventrally flattened, not extending beyond the posterior margin of the skull, and lacking any significant lateral inflation. The occipital region is gently rounded, with no prominent ridges or projections.

**Table 4.** Craniodental measurements of the *Euroscaptor* species in Vietnam. For measurements abbreviations, see Materials and methods section. The complete list of specimens included in the craniodental analysis is provided in Suppl. material 2.

Character	Species (n)					
	*E. darwini* sp. nov. (5)	*E. subanura* (21)	*E. ngoclinhensis* (4)	*E. parvidens* (13)	*E. kuznetsovi* (11)	*E. orlovi* (3)
GLS	30.51–31.01 30.88 ± 0.21	29.49–31.69 30.71 ± 0.55	31.83–33.03 32.53 ± 0.54	33.53–34.76 34.07 ± 0.41	31.75–34.72 33.74 ± 0.73	32.87–33.91 33.50 ± 0.56
BB	13.77–14.26 14.02 ± 0.22	13.44–14.67 14.01 ± 0.29	14.37–14.76 14.59 ± 0.17	14.11–15.32 14.78 ± 0.33	14.71–15.67 15.22 ± 0.32	15.22–16.04 15.51 ± 0.46
IOB	6.72–6.87 6.82 ± 0.06	6.53–7.31 6.83 ± 0.2	6.72–7.21 6.98 ± 0.2	6.41–7.01 6.78 ± 0.16	6.67–7.58 7.04 ± 0.22	7.19–7.31 7.25 ± 0.06
BIOF	5.41–5.77 5.62 ± 0.14	6.07–6.46 6.25 ± 0.27	6.16–6.46 6.36 ± 0.14	6.53–6.76 6.64 ± 0.12	6.18–6.92 6.55 ± 0.21	6.26–6.37 6.32 ± 0.06
RB	4.12–4.27 4.19 ± 0.06	3.84–4.41 4.09 ± 0.14	4.06–4.34 4.20 ± 0.12	4.28–4.74 4.58 ± 0.13	4.13–4.47 4.33 ± 0.1	4.21–4.36 4.30 ± 0.08
LZA	7.88–8.22 8.01 ± 0.13	6.83–7.74 7.33 ± 0.26	8.11–8.72 8.31 ± 0.28	8.69–9.51 9.06 ± 0.27	8.38–9.08 8.62 ± 0.36	8.18–8.39 8.31 ± 0.11
PZRW	10.09–10.37 10.21 ± 0.11	9.19–10.18 9.87 ± 0.3	10.54–10.77 10.64 ± 0.1	10.02–11.15 10.57 ± 0.29	10.53–11.32 11.03 ± 0.27	10.44–10.79 10.64 ± 0.18
ACRB	6.93–7.19 7.06 ± 0.11	7.01–7.28 7.18 ± 0.07	6.66–6.74 6.70 ± 0.04	6.39–6.54 6.46 ± 0.05	7.25–7.45 7.32 ± 0.06	6.68–6.81 6.76 ± 0.07
FMW	3.35–3.54 3.43 ± 0.07	3.27–3.75 3.53 ± 0.16	3.81–4.02 3.89 ± 0.09	3.62–4.23 3.88 ± 0.18	3.55–4.09 3.76 ± 0.17	3.45–3.92 3.68 ± 0.24
CCoL	21.41–21.88 21.59 ± 0.17	20.75–22.43 21.62 ± 0.51	23.07–23.65 23.23 ± 0.28	23.21–24.86 24.1 ± 0.41	22.68–24.38 23.96 ± 0.48	23.55–24.02 23.85 ± 0.26
PL	11.61–12.16 11.92 ± 0.23	11.31–12.6 12.05 ± 0.37	12.58–12.97 12.75 ± 0.16	12.74–13.69 13.31 ± 0.3	12.92–14.18 13.64 ± 0.34	13.31–13.65 13.52 ± 0.19
I^1^–M^3^	11.51–12.02 11.75 ± 0.23	11.19–12.72 11.81 ± 0.41	12.17–12.44 12.30 ± 0.11	12.51–13.24 12.81 ± 0.21	12.55–13.61 13.19 ± 0.34	12.85–13.21 13.03 ± 0.18
I^1^–P^4^	7.15–7.53 7.35 ± 0.16	6.73–7.79 7.28 ± 0.3	7.29–7.72 7.45 ± 0.19	7.67–8.37 7.95 ± 0.2	7.75–8.37 8.04 ± 0.21	8.05–8.19 8.12 ± 0.07
I^1^–P^3^	5.72–6.07 5.92 ± 0.14	5.16–6.06 5.7 ± 0.28	5.47–5.88 5.75 ± 0.19	5.84–6.45 6.15 ± 0.15	5.95–6.65 6.31 ± 0.21	6.27–6.54 6.40 ± 0.14
I^1^–C	2.94–3.17 3.03 ± 0.11	2.62–3.09 2.90 ± 0.15	3.02–3.21 3.11 ± 0.08	3.22–3.76 3.46 ± 0.17	3.01–3.39 3.22 ± 0.13	3.29–3.46 3.35 ± 0.09
C–M^3^	10.13–10.44 10.25 ± 0.14	9.76–11.21 10.34 ± 0.41	10.69–10.99 10.79 ± 0.14	10.63–11.58 11.07 ± 0.27	11.04–11.83 11.49 ± 0.24	11.21–11.43 11.30 ± 0.11
C–P^4^	5.51–5.93 5.69 ± 0.2	5.27–6.27 5.71 ± 0.31	5.59–5.97 5.74 ± 0.17	5.72–6.61 6.09 ± 0.25	5.89–6.45 6.18 ± 0.17	6.13–6.25 6.17 ± 0.07
P^4^–M^3^	5.91–6.18 6.10 ± 0.11	5.71–6.58 6.14 ± 0.24	6.34–6.78 6.63 ± 0.2	6.39–6.87 6.67 ± 0.12	6.61–7.21 6.93 ± 0.2	6.62–6.77 6.68 ± 0.08
P^1^–P^4^	3.99–4.11 4.06 ± 0.05	4.13–4.29 4.21 ± 0.07	4.01–4.14 4.08 ± 0.06	3.83–4.57 4.35 ± 0.21	4.44–4.87 4.58 ± 0.11	4.36–4.59 4.48 ± 0.12
M^1^–M^3^	4.54–5.03 4.75 ± 0.19	4.34–5.21 4.73 ± 0.25	4.94–5.28 5.12 ± 0.14	4.86–5.23 5.06 ± 0.1	4.79–5.62 5.24 ± 0.23	5.09–5.34 5.19 ± 0.13
M^2^–M^2^W	6.71–7.17 6.96 ± 0.22	6.65–7.81 7.06 ± 0.27	7.41–7.68 7.57 ± 0.12	7.01–7.82 7.35 ± 0.2	7.14–7.71 7.44 ± 0.19	7.27–7.59 7.44 ± 0.16
CCW	3.64–3.85 3.74 ± 0.09	3.41–3.79 3.59 ± 0.11	3.57–3.79 3.65 ± 0.1	3.79–4.41 4.03 ± 0.18	3.69–4.04 3.88 ± 0.12	3.77–3.98 3.88 ± 0.11
PWCC	2.27–2.42 2.36 ± 0.06	2.16–2.54 2.32 ± 0.1	2.31–2.49 2.40 ± 0.1	2.32–2.69 2.52 ± 0.1	2.25–2.62 2.43 ± 0.12	2.36–2.73 2.50 ± 0.2
PWM^2^M^2^	3.81–3.93 3.90 ± 0.05	3.48–4.18 3.84 ± 0.18	3.91–4.06 4.00 ± 0.07	3.67–4.21 3.93 ± 0.15	3.64–4.07 3.86 ± 0.14	3.88–3.95 3.91 ± 0.04
PWM^3^M^3^	3.91–4.06 3.98 ± 0.06	3.67–4.35 4.06 ± 0.17	4.12–4.45 4.28 ± 0.17	3.94–4.46 4.21 ± 0.15	3.79–4.43 4.21 ± 0.21	4.26–4.31 4.28 ± 0.03
ML	18.93–19.37 19.19 ± 0.16	18.33–20.28 19.24 ± 0.53	19.91–21.01 20.38 ± 0.48	20.61–22.02 21.42 ± 0.39	20.31–22.03 21.44 ± 0.44	21.04–21.46 21.21 ± 0.22
CAL	8.09–8.24 8.15 ± 0.06	7.69–8.49 8.15 ± 0.21	8.47–9.27 9.05 ± 0.38	8.61–9.53 9.24 ± 0.29	8.69–9.65 9.31 ± 0.26	9.05–9.17 9.09 ± 0.07
MH	5.73–5.84 5.78 ± 0.05	5.31–6.31 5.80 ± 0.26	6.01–6.21 6.14 ± 0.09	6.17–6.81 6.39 ± 0.19	6.03–6.81 6.49 ± 0.23	6.25–6.67 6.43 ± 0.22
i_1_–m_3_	10.88–11.45 11.16 ± 0.23	10.47–11.87 11.14 ± 0.45	11.43–11.74 11.57 ± 0.13	11.77–12.36 12.04 ± 0.18	11.86–12.94 12.5 ± 0.33	11.96–12.36 12.22 ± 0.23
p_1_–m_3_	8.99–9.43 9.21 ± 0.18	8.48–9.76 9.15 ± 0.37	9.29–9.57 9.44 ± 0.12	9.18–9.91 9.58 ± 0.18	9.74–10.55 10.23 ± 0.25	9.82–10.14 10.01 ± 0.17
p_4_–m_3_	6.07–6.47 6.28 ± 0.16	5.85–6.68 6.28 ± 0.28	6.51–6.89 6.70 ± 0.21	6.16–6.89 6.65 ± 0.2	6.72–7.47 7.14 ± 0.23	6.62–6.94 6.78 ± 0.16
i_1_–p_4_	6.11–6.27 6.21 ± 0.06	6.16–6.33 6.23 ± 0.06	6.25–6.31 6.27 ± 0.03	6.53–6.71 6.64 ± 0.06	6.78–7.06 6.93 ± 0.08	6.67–6.81 6.75 ± 0.07
m_1_–m_3_	4.89–5.29 5.05 ± 0.16	4.62–5.49 5.01 ± 0.27	4.98–5.42 5.27 ± 0.2	5.17–5.59 5.36 ± 0.1	5.27–6.18 5.68 ± 0.28	5.34–5.53 5.45 ± 0.1
i_1_–c	1.54–1.63 1.59 ± 0.04	1.65–1.88 1.76 ± 0.08	1.68–1.96 1.79 ± 0.13	1.93–2.27 2.11 ± 0.09	1.83–2.16 1.96 ± 0.11	1.74–1.81 1.77 ± 0.04
i_1_–p_1_	2.95–3.19 3.04 ± 0.09	2.77–3.34 3.09 ± 0.15	3.05–3.16 3.12 ± 0.05	3.52–3.88 3.68 ± 0.12	3.29–3.71 3.47 ± 0.14	3.37–3.54 3.48 ± 0.1
p_1_–p_4_	3.97–4.19 4.10 ± 0.09	3.63–4.39 4.00 ± 0.19	3.91–4.14 3.99 ± 0.1	3.94–4.41 4.18 ± 0.16	4.27–4.69 4.46 ± 0.13	4.41–4.58 4.49 ± 0.09

***Mandible and dentition*.** The mandible of holotype is delicate and moderately arched, with a slender ascending ramus and a weakly developed angular process. The lower border of the mandible is gently concave; the coronoid process is narrow and slightly recurved. The mandibular condyle is small, and the mandibular symphysis is short and tightly fused. The species retains the primitive fossorial mole dental formula: I3/3, C1/1, P4/4, M3/3 = 44. The first upper incisor (I^1^) slightly recurved posteriorly and has a prominent cutting edge. I^2^ and I^3^ are slightly smaller than I^1^, closely aligned, and gradually taper in height and volume. In the lower jaw, the first incisor (i_1_) is small and curves slightly forward, while i_2_ and i_3_ are smaller and aligned along the lateral mandibular toothrow. The upper canines (C^1^) are moderate in size, conical, and do not protrude strongly beyond the adjacent teeth, while the lower canines (c_1_) are similar in shape to the three lower incisors. The premolars (P^1^–P^4^ / p_1_–p_4_) are asymmetrical in size. In the upper jaw, P^2^ is the smallest of the series, whereas P^4^ is the largest and more robust in overall dimensions—nearly 2.5 times the size of P^2^. P^1^ and P^3^ are almost equal in crown height. In the lower jaw, there is a clear size progression, with p_4_ being the largest. The p_1_ and p_4_ are subequal, while p_2_ is slightly reduced. The upper molars (M^1^–M^3^) exhibit a narrow lingual margin, and instead of hypocones, they possess well-developed metaconules, especially on P^2^ and P^1^, which are clearly visible from the lingual view. M^3^ displays a triangular occlusal outline, with a prominent trigon and an anteriorly shifted protocone. The lower molars (m_1_–m_3_) lack hypoconulids, and cuspid separation is relatively weak.

***Pelvic morphology*.** The pelvic girdle is slender and reduced in overall robustness. The ischia are shortened and thin, contributing to a narrow pelvic outlet (Fig. 5). The sacral vertebrae articulate weakly with the ilia, and the pelvis shows no evidence of expanded muscle attachment sites.

##### Female secondary sexual characters.

**Figure 6.**
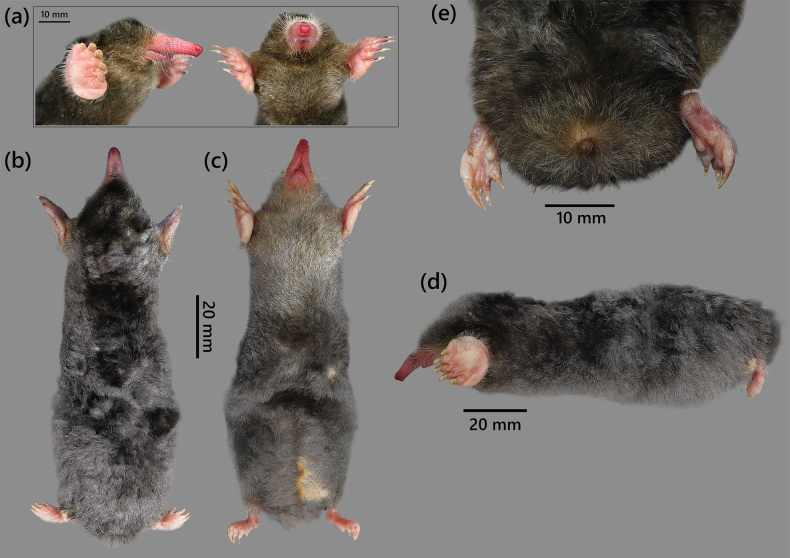
External morphology of *Euroscaptor darwini* sp. nov. a. Lateral–ventral view of partial body in laboratory; b. Dorsal view; c. Ventral view; d. Lateral view; e. Truncated–tailed of paratype specimen (NTS.2025.PL.02, ♀).

The adult female paratype (NTS.2025.PL.02) exhibits clearly identifiable sexual characteristics indicative of reproductive maturity (Fig. 6). External morphological inspection reveals the presence of two pairs of inguinal mammae, symmetrically positioned along the lower abdomen. The nipples are well-developed and slightly protruding, with the surrounding fur slightly parted. The most prominent external feature is a conspicuous pale-yellow ventral pattern, extending longitudinally from the lower thoracic region to the pelvic area. The streak is localized along the midline, forming a contrast with the darker grey–brown ventral pelage, and is absent in male and non-breeding female specimens examined in the series. This individual was confirmed to be gravid at the time of capture, based on visible abdominal distension and the presence of developing embryos upon dissection.

##### Variation.

The holotype displays a uniformly dark greyish–black dorsum with a faint silvery sheen, while the ventral surface is only marginally lighter, resulting in a nearly unicolored appearance. In contrast, female paratypes show more pronounced ventral pallor, especially in the throat and pelvic regions, sometimes accompanied by a brownish tinge around the chest. The pregnant female (NTS.2025.PL.02) has a distinct pale-yellow fur area on the mid-ventral surface. Particularly, female paratypes (*n* = 4) exhibit significantly larger craniodental dimensions than the male holotype (Fig. 5, Table 4). Female specimens show greater GLS (≤31.01 mm), maxillary toothrow length, and mandibular length in size. However, the overall dental morphology remains conserved across sexes. Variation is also apparent in tail vertebrae counts. The holotype male possesses only six caudal vertebrae, which corresponds to its externally vestigial tail. In the four female paratypes, three individuals (NTS.2025.PL.03, NTS.2025.PL.04, NTS.2025.PL.05) also exhibit six caudal vertebrae, but the pregnant female (NTS.2025.PL.02) differs in having seven (Fig. 5g).

##### Etymology.

The specific epithet *darwini* honors the eminent naturalist Charles Darwin, whose foundational contributions to evolutionary biology have profoundly influenced modern systematics and the understanding of speciation. Darwin’s insights have had a particularly strong impact on the authors of this study. We propose “Darwin’s mole” as the English common name, and “Chuột chũi Darwin” as the Vietnamese common name, reflecting the most prominent morphological trait and honoring the individual commemorated.

##### Distribution.

*Euroscaptor darwini* sp. nov. is currently known only from its type locality within Pu Luong NR, Thanh Hoa Province, north-central Vietnam. All known specimens were collected along a forested elevational transect on the southwestern ridge of Pu Luong Mountain, at altitudes ranging from 900 to 1100 meters a.s.l.

##### Ecological habitat notes.

*Euroscaptor darwini* sp. nov. was encountered in closed-canopy primary evergreen forest at the Pu Luong NR. The forest at the collection sites exhibits a dense, stratified structure with an upper canopy dominated by large dipterocarp and Fagaceae trees, an understory layer of palms, shrubs, and saplings, and a ground layer covered in deep leaf litter, decomposing organic material, and fine root networks. The soil is dark brown to reddish, loamy to clay-loam in texture, moist but well-drained, and free of surface rocks or exposed limestone. Specimens were collected along narrow animal paths, under thick vegetation, and beside moss-covered tree bases, where soil is particularly soft and cool. All trapping sites were located in non-karst areas with friable, humus-rich forest soils, and deep, organic substrates.

##### Comparisons.

The new species is distinguished from its close congeners by its truncated tail, along with distinctive cranial morphology, including a slender zygomatic arch and a narrow pelvic structure.

**Figure 7.**
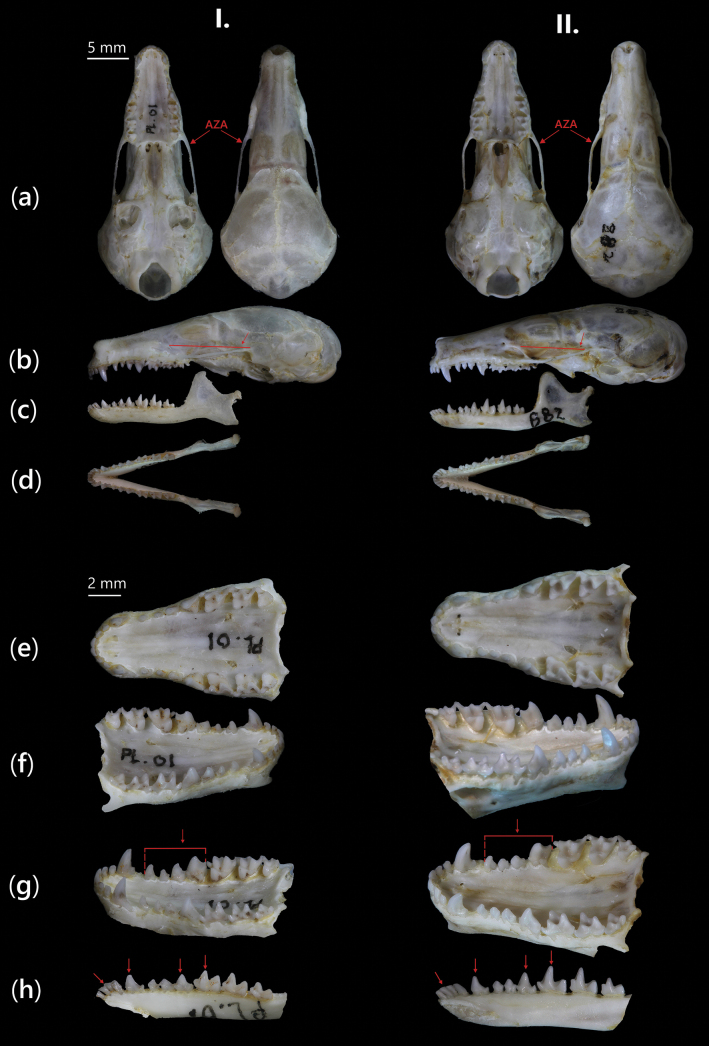
Cranium and mandible of (I) *Euroscaptor darwini* sp. nov. (Holotype, NTS.2024.PL.01, male) and (II) *E. subanura* (Holotype, SIK0882, male). From top to bottom: a. Dorsal–ventral, b, c. Lateral views of cranium and mandible; d, e. Occlusal views of mandible and cranium; f, g. Lingual view (left site and right side) of upper toothrow, respectively; h. Lingual view (right side) of lower toothrow. Distinct morphological features are labelled AZA (anterior part of the zygomatic arch) and with red arrows.

**Figure 8.**
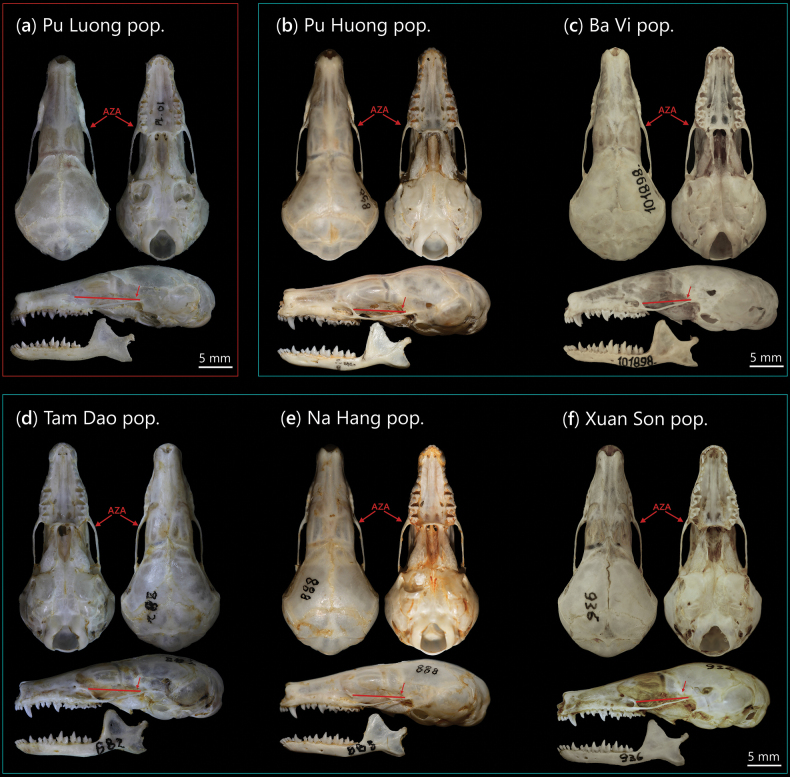
Cranium and mandible of a. *Euroscaptor darwini* sp. nov. (holotype) and five specimens representing five populations of *E. subanura*, including: b. The population closest to the new species from Pu Huong NR; c. The Ba Vi NP population; d. The type population from Tam Dao; e. The Na Hang specimen; and f. The Xuan Son NP population. Distinct morphological features are labelled with red arrows.

*Euroscaptor darwini* sp. nov. is most similar morphologically to *E. subanura*, with which it shares a markedly reduced tail length and compact body proportions. However, they differ in both craniodental morphology and external characters. Interestingly, despite the significantly narrower breadth across the interorbital foramina (BIOF: 5.41–5.77 mm in *E. darwini* vs 6.07–6.46 mm in *E. subanura*), the lateral zygomatic arch (LZA) in *E. darwini* is remarkably elongated (7.88–8.22 mm), exceeding that of *E. subanura* (6.83–7.74). This associated with differences in the shape of the zygomatic arch: in *E. darwini*, the arch is slender and tapers posteriorly toward the palatine, whereas in *E. subanura*, the anterior part zygomatic arch is slightly expanded laterally, resulting in a more parallel configuration between the left and right arches (Fig. 7). This zygomatic difference is further supported by comparative measurements across multiple *E. subanura* populations. All examined populations of *E. subanura*—from Tam Dao, Na Hang, Xuan Son, Ba Vi, and Pu Huong—exhibit consistently shorter LZA values (Fig. 8, Table 5).

**Table 5.** Craniodental measurements (in mm) of type specimens of *Euroscaptor darwini* sp. nov. (Voucher: 1♂ NTS.2024.PL.01; 4♀♀ NTS.2025.PL.02–NTS.2025.PL.05) and *E. subanura* (Voucher: 4♂♂ SIK0882, SIK0884, SIK0875, SIK0876; 1♀ SIK0883), as well as additional specimens representing three other Vietnamese populations of *E. subanura* from Pu Huong NR (Nghe An Province), Na Hang (Tuyen Quang Province), and Xuan Son NP (Phu Tho Province).

Character	*E. darwini* sp. nov.		*E. subanura*		*E. subanura*	*E. subanura*	*E. subanura*	
	Pu Luong NR (Thanh Hoa) pop.		Tam Dao (Tuyen Quang) pop.		Pu Huong NR (Nghe An) pop.	Na Hang (Tuyen Quang) pop.	Xuan Son NP (Phu Tho) pop.	
	Male (1)	Female (4)	Male (4)	Female (1)	Female (1)	Male (2)	Male (3)	Female (6)
GLS	30.51	30.94–31.01 30.97 ± 0.03	30.44–30.96 30.66 ± 0.22	31.41	31.33	30.75–31.69 31.22 ± 0.66	30.49–30.98 30.78 ± 0.26	29.96–31.02 30.48 ± 0.57
BB	13.76	13.84–14.26 14.08 ± 0.21	13.44–14.23 13.75 ± 0.34	14.44	14.33	13.93–14.67 14.30 ± 0.52	13.91–14.47 14.15 ± 0.29	13.58–14.19 13.92 ± 0.23
IOB	6.72	6.83–6.87 6.85 ± 0.02	6.57–6.86 6.77 ± 0.14	6.97	6.74	7.13–7.14 7.14 ± 0.01	6.59–7.05 6.79 ± 0.24	6.53–7.13 6.82 ± 0.21
BIOF	5.41	5.59–5.77 5.68 ± 0.09	6.13–6.21 6.17 ± 0.04	6.21	6.27	6.27–6.35 6.31 ± 0.06	6.12–6.22 6.16 ± 0.05	6.16–6.38 6.29 ± 0.08
RB	4.12	4.17–4.27 4.21 ± 0.04	3.84–4.29 4.10 ± 0.19	4.21	4.26	3.99–4.41 4.20 ± 0.3	3.96–4.16 4.03 ± 0.11	4.04–4.28 4.13 ± 0.09
LZA	8.22	7.88–8.02 7.96 ± 0.06	7.24–7.61 7.43 ± 0.16	7.68	7.63	7.01–7.54 7.28 ± 0.37	7.15–7.51 7.28 ± 0.2	7.11–7.51 7.28 ± 0.17
PZRW	10.23	10.09–10.37 10.21 ± 0.13	10.01–10.17 10.09 ± 0.08	10.13	9.85	9.96–10.18 10.07 ± 0.16	9.19–9.64 9.44 ± 0.23	9.34–10.16 9.78 ± 0.36
ACRB	6.92	6.98–7.19 7.09 ± 0.11	7.12–7.26 7.19 ± 0.07	7.29	7.18	7.27–7.35 7.31 ± 0.06	7.01–7.16 7.11 ± 0.08	7.11–7.28 7.21 ± 0.07
FMW	3.35	3.38–3.54 3.45 ± 0.07	3.27–3.71 3.46 ± 0.19	3.27	3.67	3.35–3.58 3.47 ± 0.16	3.51–3.67 3.57 ± 0.09	3.45–3.75 3.63 ± 0.13
CCoL	21.41	21.54–21.88 21.64 ± 0.16	21.42–21.83 21.68 ± 0.2	22.31	22.26	21.49–22.13 21.81 ± 0.45	20.92–21.48 21.05 ± 0.45	21.15–21.91 21.52 ± 0.42
PL	11.61	11.81–12.16 12 ± 0.17	11.55–12.53 12.03 ± 0.4	12.6	12.32	11.72–12.51 12.12 ± 0.56	11.98–12.38 12.12 ± 0.22	12.11–12.69 12.38 ± 0.31
I^1^–M^3^	11.51	11.59–12.02 11.81 ± 0.22	11.86–12.14 11.98 ± 0.12	12.72	12.16	11.89–12.33 12.11 ± 0.31	11.43–12.02 11.67 ± 0.31	11.58–12.21 11.84 ± 0.35
I^1^–P^4^	7.15	7.23–7.53 7.38 ± 0.17	7.17–7.36 7.25 ± 0.08	7.79	7.27	7.53–7.58 7.56 ± 0.04	6.73–7.68 7.21 ± 0.39	7.23–7.44 7.33 ± 0.11
I^1^–P^3^	5.92	5.72–6.07 5.92 ± 0.16	5.56–5.88 5.69 ± 0.16	6.03	5.78	5.95–6.06 6.01 ± 0.08	5.16–5.97 5.61 ± 0.35	5.68–5.95 5.85 ± 0.15
I^1^–C	3.12	2.94–3.17 3.01 ± 0.11	2.62–3.04 2.84 ± 0.17	2.92	2.99	2.97–3.09 3.03 ± 0.08	2.76–3.09 2.96 ± 0.17	2.68–3.09 2.88 ± 0.17
C–M^3^	10.13	10.14–10.44 10.28 ± 0.15	10.47–10.76 10.58 ± 0.13	11.21	10.55	10.24–10.69 10.47 ± 0.32	10.03–10.55 10.24 ± 0.28	9.76–10.76 10.17 ± 0.44
C–P^4^	5.51	5.56–5.93 5.72 ± 0.21	5.56–5.71 5.65 ± 0.07	6.27	5.49	5.63–5.79 5.71 ± 0.11	5.81–6.08 5.95 ± 0.14	5.31–6.05 5.70 ± 0.37
P^4^–M^3^	5.91	6.12–6.18 6.15 ± 0.03	6.27–6.41 6.32 ± 0.06	6.58	6.39	6.28–6.45 6.37 ± 0.12	5.81–6.11 5.92 ± 0.17	5.71–6.26 5.99 ± 0.2
P^1^–P^4^	3.99	4.02–4.11 4.07 ± 0.05	4.19–4.23 4.21 ± 0.02	4.27	4.25	4.16–4.25 4.21 ± 0.06	4.18–4.29 4.23 ± 0.06	4.13–4.29 4.21 ± 0.07
M^1^–M^3^	4.54	4.65–5.03 4.80 ± 0.17	4.81–4.98 4.92 ± 0.08	5.21	4.91	4.75–5.01 4.88 ± 0.18	4.43–4.71 4.54 ± 0.15	4.34–4.87 4.55 ± 0.23
M^2^–M^2^W	6.69	6.72–7.17 7.02 ± 0.2	7.01–7.33 7.12 ± 0.14	7.81	7.14	7.02–7.26 7.14 ± 0.17	6.71–7.06 6.86 ± 0.18	6.65–7.29 6.96 ± 0.22
CCW	3.71	3.64–3.85 3.75 ± 0.1	3.57–3.71 3.64 ± 0.07	3.73	3.74	3.71–3.76 3.74 ± 0.04	3.49–3.61 3.55 ± 0.06	3.41–3.79 3.56 ± 0.13
PWCC	2.41	2.27–2.42 2.35 ± 0.06	2.17–2.37 2.31 ± 0.09	2.41	2.54	2.31–2.36 2.34 ± 0.04	2.29–2.33 2.31 ± 0.02	2.16–2.47 2.29 ± 0.13
PWM^2^M^2^	3.91	3.81–3.93 3.90 ± 0.06	3.57–3.96 3.80 ± 0.16	4.15	3.95	3.95–3.99 3.97 ± 0.03	3.69–3.89 3.80 ± 0.1	3.56–3.94 3.83 ± 0.14
PWM^3^M^3^	3.91	3.92–4.06 3.99 ± 0.06	3.92–4.09 4.03 ± 0.07	4.35	4.11	4.07–4.21 4.14 ± 0.1	3.67–4.19 3.98 ± 0.27	3.91–4.17 4.07 ± 0.12
ML	18.93	19.21–19.37 19.26 ± 0.07	19.1–19.47 19.32 ± 0.17	20.28	19.77	19.07–20.18 19.63 ± 0.78	18.33–19.86 18.89 ± 0.55	18.91–19.69 19.35 ± 0.4
CAL	8.14	8.09–8.24 8.15 ± 0.07	8.05–8.33 8.15 ± 0.12	8.33	8.29	7.81–8.29 8.05 ± 0.34	7.69–8.36 8.05 ± 0.27	8.16–8.41 8.31 ± 0.13
MH	5.84	5.73–5.83 5.76 ± 0.05	5.72–6.01 5.81 ± 0.13	6.03	6.26	5.64–6.08 5.86 ± 0.31	5.53–6.07 5.76 ± 0.19	5.78–6.31 6.00 ± 0.28
i_1_–m_3_	10.88	11.01–11.45 11.23 ± 0.19	10.69–11.57 11.18 ± 0.37	11.87	11.67	11.38–11.59 11.49 ± 0.15	11.05–11.33 11.19 ± 0.14	10.51–11.65 10.95 ± 0.46
p_1_–m_3_	8.99	9.16–9.43 9.23 ± 0.21	9.24–9.39 9.32 ± 0.08	9.76	9.56	9.21–9.52 9.37 ± 0.22	8.63–9.54 8.99 ± 0.34	9.03–9.31 9.17 ± 0.14
p_4_–m_3_	6.07	6.22–6.47 6.33 ± 0.13	6.39–6.54 6.46 ± 0.07	6.67	6.68	6.26–6.48 6.37 ± 0.16	5.91–6.28 6.14 ± 0.2	5.85–6.49 6.13 ± 0.25
i_1_–p_4_	6.11	6.21–6.27 6.24 ± 0.03	6.22–6.29 6.25 ± 0.03	6.24	6.19	6.32–6.37 6.35 ± 0.04	6.16–6.24 6.21 ± 0.04	6.17–6.32 6.21 ± 0.06
m_1_–m_3_	4.89	4.97–5.29 5.09 ± 0.15	5.15–5.25 5.20 ± 0.04	5.34	5.49	5.09–5.29 5.19 ± 0.14	4.68–5.06 4.84 ± 0.2	4.62–5.28 4.84 ± 0.25
i_1_–c	1.54	1.56–1.63 1.60 ± 0.03	1.72–1.81 1.74 ± 0.09	1.86	1.88	1.77–1.79 1.78 ± 0.01	1.68–1.86 1.74 ± 0.1	1.69–1.88 1.77 ± 0.07
i_1_–p_1_	2.98	2.95–3.19 3.06 ± 0.1	3.05–3.17 3.10 ± 0.05	3.02	3.22	3.34–3.39 3.37 ± 0.04	2.77–3.22 3.05 ± 0.18	3.11–3.17 3.13 ± 0.03
p_1_–p_4_	3.97	4.07–4.19 4.10 ± 0.1	3.87–4.12 3.99 ± 0.11	4.15	3.94	4.08–4.12 4.10 ± 0.03	3.79–4.23 3.98 ± 0.16	3.93–4.25 4.1 ± 0.16

The *E. darwini* displays shorter crown heights and weaker cuspid development, particularly on p_4_, m_1_, m_2_, m_3_. In dorsal view, the rostral portion of *E. darwini* appears narrower and more tapered anteriorly, with the interorbital region slightly more constricted. The snout breadth and palatal length are both consistently narrower in *E. darwini*, showing a slender craniofacial profile (Figs 7, 8). This is supported by the shorter maxillary premolar length (P^1^–P^4^: 3.99–4.11 mm in *E. darwini* vs 4.13–4.29 mm in *E. subanura*) and the compact mandibular incisor row length (i_1_–c: 1.54–1.63 mm in *E. darwini* vs 1.65–1.88 mm in *E. subanura*) (Table 4). Furthermore, dental spacing and size differentiation are pronounced: I^1^ in *E. darwini* is medium size and curved, but smaller than that of *E. subanura*, and the spacing between I^2^ and I^3^ is more compressed. The lower incisors and premolars also follow a progressive enlargement posteriorly, but are overall less massive and vertically developed in *E. darwini*, contributing to a reduced masticatory apparatus.

In terms of external measurements, *E. darwini* displays a shorter tail than *E. subanura*, with tail lengths ranging from 2.11–3.74 mm compared to 2.5–5.0 mm as reported by Kawada (2016) and Bui (2022). This difference occurs despite overlapping head–body lengths: 115.71–127.93 mm in *E. darwini* vs 113.0–131.5 mm and 107.0–130.5 mm in *E. subanura* (Kawada 2016, Bui 2022), resulting in a lower tail to body length ratio (1.82–2.92%) compared to *E. subanura* (1.96–4.24% and 2.33–3.85%) (Table 3). This external truncation is supported by osteological evidence: *E. darwini* possesses only six or seven caudal vertebrae, whereas *E. subanura* retains nine or ten.

*Euroscaptor darwini* possesses an overall smaller skull compared to *E. ngoclinhensis*, as reflected by shorter measurements in GLS (30.51–31.01 mm vs 31.83–33.03 mm), LZA (7.88–8.22 mm vs 8.11–8.72 mm), and mandibular length (ML: 18.93–19.37 mm vs 19.91–21.01 mm). It also has a narrower interorbital breadth (BIOF: 5.41–5.77 mm vs 6.16–6.46 mm) and a shorter lower post third-premolar toothrow (p_4_–m_3_: 6.07–6.47 mm vs 6.51–6.89 mm). The upper toothrow (I^1^–M^3^) is reduced in length (11.51–12.02 mm in *E. darwini* vs 12.17–12.44 mm in *E. ngoclinhensis*, and up to 13.0 mm in *E. parvidens* and *E. kuznetsovi*). The distance between lower incisor-canine (i_1_–c) is also shorter (1.54–1.63 mm), representing the smallest value recorded within the genus.

Compared to *E. parvidens*, the cranium of *E. darwini* is more slender, with a narrower basal breadth and reduced palatal length. GLS is significantly shorter (30.51–31.01 mm vs 33.53–34.76 mm), BB is narrower (13.77–14.26 mm vs 14.11–15.32 mm), and the width between upper canine CCW is reduced (3.64–3.85 mm vs 3.79–4.41 mm). The mandible is likewise smaller (ML: 18.93–19.37 mm vs 20.61–22.02 mm), with shortened m_1_–m_3_ (4.89–5.29 mm vs 5.17–5.59 mm), i_1_–p_1_ (2.95–3.19 mm vs 3.52–3.88 mm) segments, yielding a more compact jaw proportion.

*Euroscaptor darwini* differs from *E. orlovi* and *E. kuznetsovi* in having a smaller cranium, shorter palatal length (PL: 11.61–12.16 mm vs 13.31–13.65 mm in *E. orlovi*), and lesser posterior zygomatic root width (PZRW: 10.09–10.37 mm vs 10.44–10.79 mm). These proportions result in a noticeably more compact and domed neurocranial profile in dorsal view. Furthermore, the upper molars of *E. darwini* are less transversely expanded than those of *E. kuznetsovi*, as indicated by a narrower M^2^–M^2^ width (6.71–7.17 mm in *E. darwini* vs ≤ 7.8 mm in *E. kuznetsovi*). The mandibular symphysis in *E. darwini* is relatively shorter and more vertically aligned, contributing to a blunter jaw profile compared to the more elongated mandibles seen in larger *Euroscaptor*.

### ﻿Cranial morphometrics and size differences in *Euroscaptor darwini* sp. nov.

**Figure 9.**
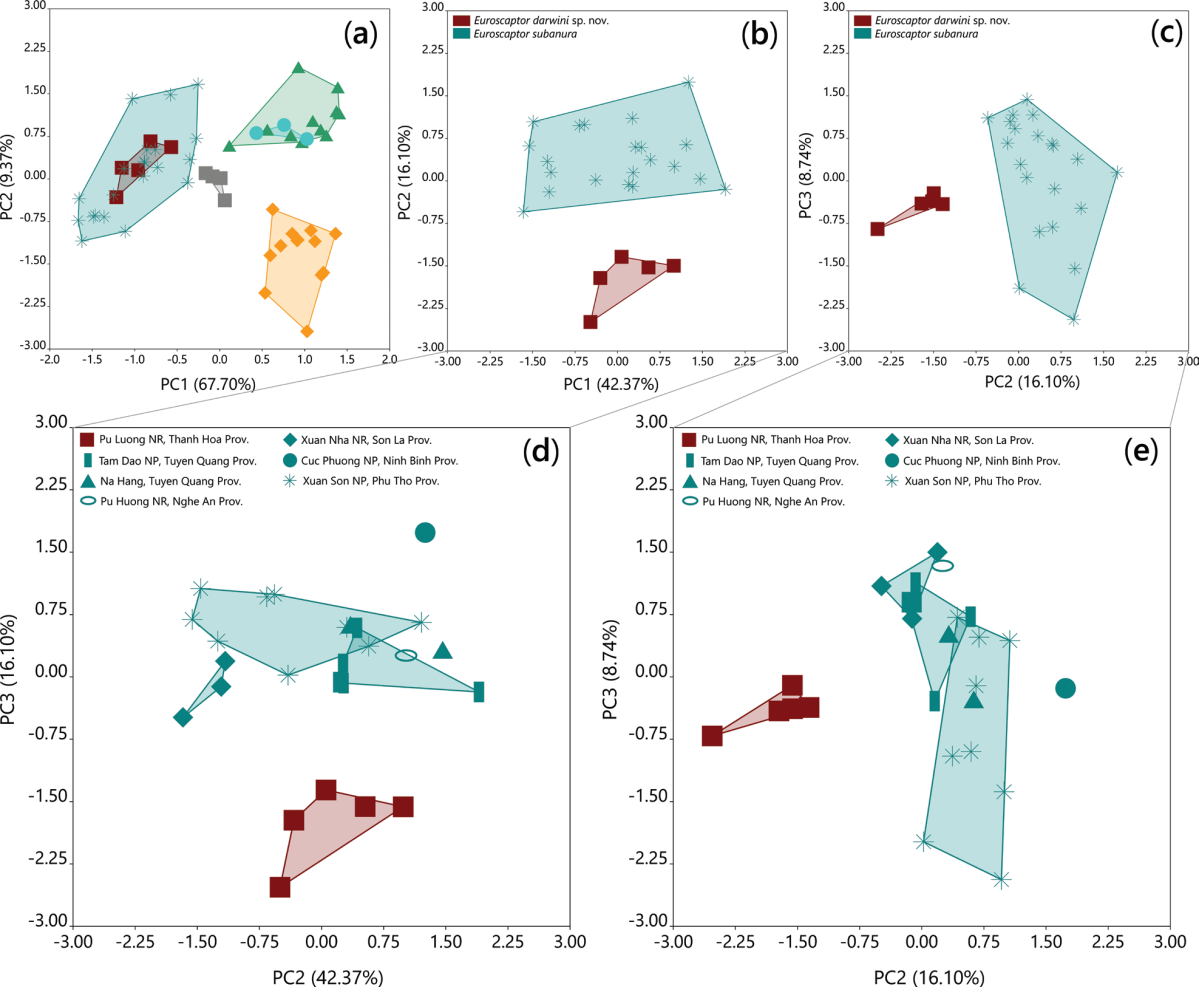
Bivariate scatterplots of the 1^st^, 2^nd^, and 3^rd^ principal component scores derived from log-transformed craniodental measurements for *Euroscaptor* species in Vietnam. a. Scatterplot of PC1 vs PC2 showing differentiation among *E. darwini* (red squares), *E. subanura* (asterisks), *E. kuznetsovi* (triangles), *E. orlovi* (circles), *E. ngoclinhensis* (gray squares), and *E. parvidens* (diamonds); b, c. Plot of PC1 vs PC2 and PC2 vs PC3 for *E. darwini* (red color) and *E. subanura* (teal color); d, e. Plot of PC1 vs PC2 and PC2 vs PC3 for *E. darwini* (red color) and six *E. subanura* populations in Vietnam (teal color).

Principal Component Analysis (PCA) of 36 log-transformed craniodental measurements revealed morphometric differentiation in all Vietnamese *Euroscaptor* taxa (Fig. 9a). The differences among taxa were detected by one-way ANOVA (P < 0.05) with factor loadings for each variable presented in Table 6. PC1, accounting for 67.70% of the total variance, primarily reflects overall size differences, especially in mandibular, maxillary lengths and toothrow proportions. PC2 contributes an additional 9.37% of the variation. *Euroscaptor darwini* and *E. subanura* form a tight and completely overlapping cluster in the negative PC1 space. No significant separation is observed between them along PC1 (p = 0.784). *Euroscaptor ngoclinhensis* occupies a more central position along PC1 and is clearly separated from the *E. darwini*–*E. subanura* cluster. PC1 scores of *E. darwini* are significantly lower than those of larger-bodied taxa, including *E. parvidens*, *E. orlovi*, and *E. kuznetsovi* (One-way ANOVA, P < 0.01). *E. parvidens* forms a discrete cluster in PC1 and PC2 quadrant, significantly separated from both *E. orlovi* and *E. kuznetsovi* (P < 0.01). Meanwhile, *E. orlovi* and *E. kuznetsovi* exhibit partial overlap both in PC1 and PC2 (t-test, t = 1.204, p = 0.237), but remain distinct from the smaller-bodied *E. darwini*–*E. subanura* group.

**Table 6.** Character factor loadings for log-transformed measurements (PCs 1, 2, 3) among all taxa and between *E. darwini* sp. nov. and *E. subanura*.

Character	All taxa			*E. darwini* and *E. subanura*		
	PC 1	PC 2	PC 3	PC 1	PC 2	PC 3
GLS	0.17	–0.05	–0.05	0.09	–0.03	0.01
BB	0.12	0.04	–0.06	0.06	0.00	0.06
IOB	0.04	0.13	0.01	0.08	0.05	0.01
BIOF	0.09	0.02	0.24	–0.01	0.44	0.17
RB	0.14	–0.17	–0.16	0.10	–0.17	0.19
LZA	0.27	–0.24	–0.35	0.05	–0.45	0.00
PZRW	0.14	0.06	–0.18	0.06	–0.18	0.17
ACRB	–0.07	0.32	0.17	0.02	0.08	0.05
FMW	0.14	–0.19	0.00	–0.04	0.18	–0.07
CCoL	0.18	–0.02	–0.08	0.12	–0.01	0.02
PL	0.20	0.08	0.01	0.12	0.02	–0.05
I^1^–M^3^	0.18	0.12	0.02	0.21	0.03	0.07
I^1^–P^4^	0.17	0.08	0.10	0.22	0.03	–0.22
I^1^–P^3^	0.17	0.13	0.11	0.26	–0.08	–0.37
I^1^–C	0.23	–0.30	–0.12	0.10	–0.21	–0.07
C–M^3^	0.17	0.17	0.01	0.23	0.05	0.03
C–P^4^	0.15	0.13	0.15	0.24	0.11	–0.38
P^4^–M^3^	0.18	0.13	–0.09	0.19	0.00	0.31
P^1^–P^4^	0.11	0.12	0.25	0.02	0.15	–0.36
M^1^–M^3^	0.17	0.19	–0.14	0.29	–0.03	0.29
M^2^–M^2^W	0.10	0.06	–0.11	0.18	0.06	0.14
CCW	0.16	–0.13	–0.14	0.11	–0.18	0.04
PWCC	0.12	–0.12	–0.16	0.06	–0.10	0.13
PWM^2^M^2^	0.04	0.00	–0.11	0.18	0.01	0.11
PWM^3^M^3^	0.09	–0.02	–0.04	0.13	0.15	0.21
ML	0.19	0.01	–0.06	0.15	–0.02	0.01
CAL	0.22	–0.04	–0.16	0.07	–0.04	0.05
MH	0.20	0.02	–0.06	0.19	0.00	–0.10
i_1_–m_3_	0.18	0.13	0.06	0.23	0.04	–0.06
p_1_–m_3_	0.15	0.24	0.01	0.24	0.01	–0.04
p_4_–m_3_	0.17	0.25	–0.08	0.25	–0.01	0.14
i_1_–p_4_	0.14	0.07	0.09	0.04	0.04	–0.01
m_1_–m_3_	0.18	0.22	–0.04	0.31	–0.04	0.20
i_1_–c	0.26	–0.39	0.59	0.02	0.55	0.08
i_1_–p_1_	0.27	–0.19	0.30	0.21	0.18	–0.25
p_1_–p_4_	0.15	0.27	0.10	0.24	0.02	–0.28
% variance	67.70	9.37	4.12	42.37	16.10	8.74

To clarify the morphological divergence between *Euroscaptor darwini* and its closest relative *E. subanura*, PCA analyses were conducted using only specimens of these two species (Fig. 9b–e). The first three PCs explain 67.57% of the total variance, with PC1 accounting 42.73%, PC2: 16.10%, and PC3: 8.74% (Table 6). These components collectively reveal a pattern of subtle yet consistent differentiation between the two taxa. PC1, which reflects overall cranial size and proportions, is primarily influenced by variables associated with premolar and molar row lengths: C–P^4^ (0.24), M^1^–M^4^ (0.29), I^1^–P^3^ (0.26), m_1_–m_3_ (0.31), and p_4_–m_3_ (0.25). PC2 is most influenced by BIOF (0.44) and i_1_–c (0.55) pointing to differences in infraorbital foramina breadth and mandibular incisor spacing. The negative loading of LZA (–0.45) further highlights variation in zygomatic arch length. PC3, which accounts for 9.07% of the variance and contributes to additional separation among taxa, is dominated by C–P^4^ (–0.38), I^1^–P^3^ (–0.37), P^4^–M^3^ (0.31) and P^1^–P^4^ (–0.36), with the latter contributing negatively to the axis and reflecting differences in premolar spacing and alignment.

**Figure 10.**
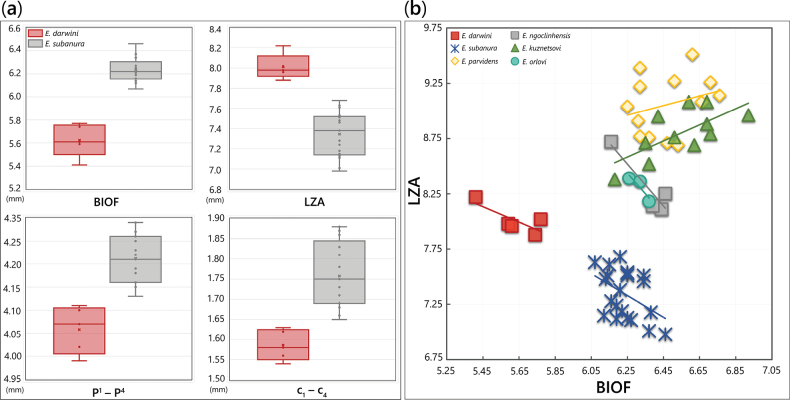
a. Boxplots showing range (minimum to maximum, mean value indicated by horizontal lines), interquartile range (represented by the rectangular box) of the most contributing measurements to high factor loadings across multiple PCs; b. Plots of the breadth between infraorbital foramina (BIOF) against the inner length of zygomatic arch (LZA) for the genus *Euroscaptor* in Vietnam.

Multivariate analysis provides quantitative support for the morphological distinctiveness of *E. darwini* relative to *E. subanura*. Among the variables contributing most strongly to species separation, length of the zygomatic arch (LZA) and breadth between the infraorbital foramina (BIOF) present the most pronounced divergence. Specimens of *E. darwini* consistently display a more elongated zygomatic arch (LZA: 7.88–8.22 mm) and noticeably narrower interorbital breadth (BIOF: 5.59–5.77 mm in females, 5.41 mm in the male) compared to *E. subanura* (BIOF: 6.17–6.21 mm in males, 6.21 mm in the female; Table 5, Fig. 10a, b). In the bivariate plot of BIOF against LZA (Fig. 10b), although BIOF remains relatively consistent across most *Euroscaptor* species in Vietnam, *E. darwini* exhibits a distinctly lower value for this trait. This deviation results in a conspicuous leftward shift of the *E. darwini* cluster in the plot (Fig. 10b), clearly separating it from all other congeners.

Several variables including C–P^4^, I^1^–P^3^, I^1^–M^3^, P^1^–P^4^, and P^4^–M^3^, constantly indicated high loadings across multiple PCs (Table 6) and were significantly smaller in *E. darwini*, reflecting its relatively reduced and compact cranial and dental architecture. Pairwise character comparisons further corroborate these findings (Table 7), which demonstrate significant differentiation between *E. darwini* and *E. subanura* in nearly all dental measurements, indicating both cranial and mandibular divergence observed in the PCA results. Besides *E. subanura*, *E. darwini* also differs from other *Euroscaptor* congeners in key variables such as BIOF, LZA, CAL and I^1^–C (Table 7).

**Table 7.** Measurement variables showing significant pairwise differences between *Euroscaptor darwini* and other *Euroscaptor* species in Vietnam, based on Tukey’s test for the most influential loadings on PC1–PC3.

*E. darwini* sp. nov.	Character	*E. subanura*	*E. ngoclinhensis*	*E. parvidens*	*E. kuznetsovi*	*E. orlovi*
	Cranium	BIOF, LZA	BIOF, LZA, CAL, FMW	BIOF, LZA, CAL	BIOF, LZA, CAL, PL	BIOF, CAL, PL, MH, FMW
	Maxillary dentition	P^1^–P^4^, C–P^4^, P^4^–M^3^, C–M^3^, M^1^–M^3^	P^1^–P^4^, PWCC,	P^1^–P^4^, C–P^4^, I^1^–C, CCW, PWCC, PWM^2^M^2^, PWM^3^M^3^	I^1^–C, CCW, PWCC, PWM^2^M^2^, PWM^3^M^3^	P^1^–P^4^, C–P^4^, I^1^–C, I^1^–M^3^, M^1^–M^3^, M^2^–M^2^W, CCW, PWCC
	Mandibular dentition	i_1_–c, m_1_–m_3_, p_4_–m_3_, p_1_–m_3_, p_1_–p_4_	i_1_–c, m_1_–m_3_, p_1_–p_4_	i_1_–p_1_	i_1_–c, m_1_–m_3_, p_4_–m_3_, i_1_–p_1_	i_1_–c

In addition to interspecific differences, the female paratypes exhibit larger values across multiple cranial and external characters than the holotype male (see Table 5). A similar trend is observed in type specimens of *E. subanura* in Tam Dao, where the sole female has larger values in most cranial variables than the male series (GLS: 31.41 mm vs 30.44–30.96 mm; M^1^–M^3^: 5.21 mm vs 4.81–4.98 mm and ML: 20.28 vs 19.1–19.47 mm).

## ﻿Discussion

The genus *Euroscaptor* is known for its external morphological conservatism, a typical consequence of convergent adaptation to subterranean life (Partha et al. 2017; Sansalone et al. 2019; Biltueva et al. 2023). Nevertheless, detailed analyses of cranial, dental, and karyological traits have revealed considerable interspecific variation (Kawada 2016). In Vietnam, the distribution of *Euroscaptor* species is strongly structured by altitude, with taxa such as *E. subanura* occupying lowland habitats (< 600 m), while others (*E. orlovi*, *E. kuznetsovi*) are restricted to higher elevations (> 1000 m) (Zemlemerova et al. 2016; Bui et al. 2020a).

These altitudinal segregations coincide with geographic boundaries that may contribute to reproductive isolation, leading to divergent evolutionary trajectories across terrain and fragmented montane habitats and ultimately facilitating speciation (Kawada et al. 2009; Feijó et al. 2019; Pujolar et al. 2022; Okabe and Motokawa 2024).

### ﻿Phylogeographic isolation and divergence in *Euroscaptor darwini*

The phylogenetic analysis (Fig. 4) indicates *Euroscaptor darwini* as a distinct and well-supported lineage, clearly diverged from its closest relative *E. subanura*. With the representative by slightly longer internal branch leading to *E. darwini*, which combined with their contrasting elevational distributions, it suggests a period of independent evolution among these two species, which can be driven by ecological isolation across altitudinal gradients.

This situation aligns with montane mole speciation in Vietnam, that sharp elevational gradients and fragmented habitats can limit dispersal, reported by Shinohara et al. (2014, 2015), emphasizing the role of elevation and geographic isolation in driving species diversification. Otherwise, Zemlemerova et al. (2016) demonstrated that the Red River acts as a barrier separating genetically distinct lineages: *E. orlovi* and *E. kuznetsovi*. Comparable patterns have been observed in the genus *Talpa*, where species in *Talpa* are restricted to narrow, non-overlapping ranges with little evidence of gene flow across geographic barriers (Bannikova et al. 2015a). This can be occurred due to biological traits and behaviors such as low dispersal ability, high territoriality, and fossorial specialization, which strengthen geographic isolation. Morphological conservatism further conceals this cryptic diversity, which is only revealed through molecular phylogenetics. These studies support allopatric divergence as the primary driver of mole speciation in Vietnam, formed by topographic complexity and ecological barriers. The divergence of *E. darwini* is hypothesized as a consequence of isolation in the upland forests of central Vietnam, a region characterized by steep elevational gradients.

The idea that geographic isolation drives the divergence of populations into separate species was first systematically developed by Ernst Mayr (Mayr 1942), who first introduced the concept of allopatric speciation. His work pointed to the role of natural barriers, such as mountains, rivers, and fragmented habitats, as major factors in limiting gene flow and allowing the divergence of populations through mechanisms like genetic drift, natural selection, and local adaptation. The conceptual foundation, however, was laid earlier by Charles Darwin, through his observations of Galápagos finches (Darwin 1839, 1859), showing that the diversification of these birds across islands provided compelling evidence of geographic differentiation as the driver of speciation. In this study, our findings suggest that both geographic and ecological isolation may have contributed to the separation of *E. darwini* from closely related species. The Pu Luong type locality features a heterogeneous landscape with both karstic and non-karstic terrain, is distributed across different elevation gradients and abrupt topographic changes. *Euroscaptor darwini* was collected exclusively in a relatively flat forested patch ascending a steep escarpment exceeding 110 meters in elevation, with an estimated slope of 75–85° (Fig. 2e). This noticeable topographic discontinuity may serve as a natural barrier that isolates the *E. darwini* population from *E. subanura* populations distributing in the lower foothills, potentially initiating allopatric speciation and promoting reproductive isolation over evolutionary timescales.

### ﻿Morphological differentiation in *Euroscaptor darwini*

The morphological distinctiveness of *E. darwini* is particularly evident in the combination of two metrics: BIOF and LZA. These features are not only taxonomically informative but may also reflect functional differentiation. The geometry of the cranial lateral wall, including the configuration of the zygomatic arches, plays an important role in biomechanical factors such as bite force efficiency, attachment of masticatory muscles, and spatial organization of sensory structures, including the orbit and auditory bulla (Herring 2007; Smith and Grosse 2016; Sharp et al. 2023). In *E. darwini*, the zygomatic arches taper posteriorly toward the palate, in contrast to populations of *E. subanura* recorded in Vietnam, including the *E. subanura* population closest to the new species, collected from Pu Huong NR, Nghe An Province, in which the zygomatic arches are more developed and anteriorly expanded, forming a nearly parallel-sided cranial axis. These differences may be affected by evolutionary divergence, but they could also be influenced by the separate elevational distributions of the two species: *E. subanura* is restricted to lowland elevation below 600 m (Kawada et al. 2012; Bui 2022), whereas *E. darwini* is distributed at altitudes ranging between 900 and 1000 m a.s.l. These contrasting cranial constructions may represent adaptations to different ecological habitats associated with altitudinal segregation.

Moreover, the plot results suggests that the ratio between BIOF and LZA may serve as an informative measurement for diagnosing *E. darwini*. This ratio shows an aspect of cranial geometry that is not readily apparent from raw measurements. Comparative analyses indicate that *E. darwini* exhibits the lowest BIOF/LZA ratio among Vietnamese *Euroscaptor*, reflecting its narrow rostral region relative to the anterior elongation of zygomatic arches. Although this ratio remains relatively constant across congeners (Fig. 10b), it is significantly separate in *E. darwini* and may prove useful as a rapid diagnostic trait in morphometric analyses or initial identification. The differentiations in skull shape are also revealed through PCA involving key traits such as premolars row length, interorbital breadth, zygomatic arch length and incisors length, which may have been caused by the role of geographic and ecological separation in shaping divergent morphotypes. Within *E. darwini* population, its restriction to an upland area separated by steep cliffs from the lowland range of *E. subanura*, may be one of factors contributing to the isolation that led to the separation of two species.

In addition to craniodental differentiation, the truncated tail of *E. darwini* provides further support for this species distinctiveness. The reduction of caudal vertebrae represents a defining autapomorphy of this species, setting it apart from all other *Euroscaptor*. This trait appears consistent across individuals regardless of minor internal variation. The gravid female paratype is found to possess seven caudal vertebrae which is one more than the holotype male. Nevertheless, this anatomical difference does not manifest as any appreciable external elongation; the tail remains externally vestigial in all examined specimens (2.11–3.74 mm). While there may be slight intraspecific variation in vertebral count, it does not diminish the diagnostic value of tail reduction as a morphological identification. The persistence of a truncated tail across individuals further underscores its taxonomic utility and potential adaptive significance, possibly relating to fossorial specialization or to dispersal limitations imposed by the montane habitat.

### ﻿Reproductive biology and sexual size differences

Besides its biogeographic distinctiveness, *E. darwini* provides insights into reproductive biology. After we practice abdominal palpation and subsequent dissection gently, a single conceptus was observed in the right uterine horn. The embryo displayed distinguishable head, trunk, four limb buds, and tail bud structures, indicative of late-stage pregnancy. Although only one embryo was detected, the presence of two pairs of inguinal mammae suggests a potential litter size of two to four offspring. Additionally, the existence of the pale-yellow ventral streak in the pregnant female can function as a visual reproductive signal, probably caused by glandular secretions or hormonal fluctuations related to pregnancy or lactation. This study presents the first information of reproductive condition in *Euroscaptor darwini* sp. nov., with mating and gestation occurring during the late dry to early rainy season (April). The timing also supports the breeding season in this mole may begin as early as April, as no signs of testicular development were observed in a male *E. darwini* collected in November. This aligns with reproductive studies reported in Japanese moles (*Mogera* spp.), when a noteworthy increase in testis size has been observed from April onward, and the smallest testis length observed from November to December, indicating that breeding season likely occurs in late spring (Abe 1967).

Sexual size dimorphism may be present in *Euroscaptor darwini*, as indicated by morphometric data showing that female paratypes consistently possess larger body sizes, longer skulls, broader braincases, and generally greater dimensions across a range of cranial and external characteristic compared to the holotype male (Tables 3, 5). These differences, in combination with reproductive evidence, suggest the presence of female-biased size dimorphism in the Pu Luong population. This recurrent feature in both species may result from selective pressures favoring increased body size in females, potentially associated with greater reproductive or higher energetic requirements during gestation. However, due to limited sample sizes, particularly by the presence of sole male, additional data are needed to confirm the consistency and extent of this pattern across populations and seasons.

## ﻿Conclusions

The integration of morphological, and phylogenetic evidence firmly establishes *Euroscaptor darwini* as a distinct species. Its reproductive seasonality, potential female-biased size dimorphism, and caudal vertebrae reduction provide understanding for exploring adaptive evolution of fossorial mole. This present study indicates tail morphology acting as a taxonomically informative character and raises questions about the ecological and evolutionary pressures leading to caudal reduction in subterranean mammals. Given the functional role of tail structure in many moles, the truncated tail observed in *E. darwini* may reflect specialized burrowing behavior or adaptation to local geological substrates in Pu Luong that need further behavioral and biomechanical investigation.

The discovery of this new species highlights the underappreciated biodiversity of Vietnam’s upland ecosystems. As integrative taxonomic approaches continue to reveal cryptic diversity within the genus *Euroscaptor* and family Talpidae, the urgency of recognizing and conserving micro-endemic species like *E. darwini* becomes increasingly necessary, particularly within Pu Luong NR, where the population is experiencing decline due to anthropogenic pressures associated with ecotourism development.

## Supplementary Material

XML Treatment for Euroscaptor darwini

## ﻿Additional information

### Use of AI

No use of AI was reported.

### Author ORCIDs

Son Truong Nguyen https://orcid.org/0000-0003-3214-4407

Hai Tuan Bui https://orcid.org/0000-0003-4065-7229

Vinh Quang Dau https://orcid.org/0000-0002-8976-1644

Yen Huong Vu https://orcid.org/0009-0001-4643-167X

### Data availability

All of the data that support the findings of this study are available in the main text or Supplementary Information.

## Supplementary materials

Supplementary material 1 Morphological Data type docx Explanation note List of craniodental measurements used in this study. https://binary.pensoft.net/file/1435347This dataset is made available under the Open Database License (http://opendatacommons.org/licenses/odbl/1.0/). The Open Database License (ODbL) is a license agreement intended to allow users to freely share, modify, and use this Dataset while maintaining this same freedom for others, provided that the original source and author(s) are credited.Son Truong Nguyen, Hai Tuan Bui, Vinh Quang Dau, Phuong Dinh Le, Yen Huong Vu

Supplementary material 2 List of skull specimens, morphological Data type docx Explanation note List of *Euroscaptor* skull specimens from Vietnam examined in this study. https://binary.pensoft.net/file/1435348This dataset is made available under the Open Database License (http://opendatacommons.org/licenses/odbl/1.0/). The Open Database License (ODbL) is a license agreement intended to allow users to freely share, modify, and use this Dataset while maintaining this same freedom for others, provided that the original source and author(s) are credited.Son Truong Nguyen, Hai Tuan Bui, Vinh Quang Dau, Phuong Dinh Le, Yen Huong Vu

Supplementary material 3 List of Cyt b sequences, phylogenetic Data type docx Explanation note The list of Cyt *b* sequences used for pairwise comparisons. https://binary.pensoft.net/file/1435349This dataset is made available under the Open Database License (http://opendatacommons.org/licenses/odbl/1.0/). The Open Database License (ODbL) is a license agreement intended to allow users to freely share, modify, and use this Dataset while maintaining this same freedom for others, provided that the original source and author(s) are credited.Son Truong Nguyen, Hai Tuan Bui, Vinh Quang Dau, Phuong Dinh Le, Yen Huong Vu

Supplementary material 4 Phylogenetic Data type xlsx Explanation note The raw p–distance matrix of Cyt b sequences used for comparisons between *E. darwini* and *E. subanura* population. https://binary.pensoft.net/file/1435350This dataset is made available under the Open Database License (http://opendatacommons.org/licenses/odbl/1.0/). The Open Database License (ODbL) is a license agreement intended to allow users to freely share, modify, and use this Dataset while maintaining this same freedom for others, provided that the original source and author(s) are credited.Son Truong Nguyen, Hai Tuan Bui, Vinh Quang Dau, Phuong Dinh Le, Yen Huong Vu

Supplementary material 5 List of DNA accession number, phylogenetic, genomic Data type docx Explanation note *uroscaptor* specimens with DNA Accession Number used in the Phylogenetic analyses based on concatenated mitochondrial Cyt *b* and 12S rRNA gene sequences. Asterisks indicate holotype (*) and hash sign indicate paratype (#) specimens of both *E. darwini* and *E. subanura*. https://binary.pensoft.net/file/1435351This dataset is made available under the Open Database License (http://opendatacommons.org/licenses/odbl/1.0/). The Open Database License (ODbL) is a license agreement intended to allow users to freely share, modify, and use this Dataset while maintaining this same freedom for others, provided that the original source and author(s) are credited.Son Truong Nguyen, Hai Tuan Bui, Vinh Quang Dau, Phuong Dinh Le, Yen Huong Vu

## References

[B1] Abe H (1967) Classification and biology of Japanese Insectivora (Mammalia). II. Biological aspects. Journal of the Faculty of Agriculture, Hokkaido University 55: 429–458.

[B2] Asahara M Kryukov A Motokawa M (2012) Dental anomalies in the Japanese mole *Mogera wogura* from northeast China and the Primorsky region of Russia. Acta Theriologica 57(1): 41–48. 10.1007/s13364-011-0050-0

[B3] Badgley C Smiley TM Terry R Davis EB DeSantis LRG Fox DL Hopkins SSB Jezkova T Matocq MD Matzke N McGuire JL Mulch A Riddle BR Roth VL Samuels JX Strömberg CAE Yanites BJ (2017) Biodiversity and topographic complexity: Modern and geohistorical perspectives. Trends in Ecology & Evolution 32(3): 211–226. 10.1016/j.tree.2016.12.010 28196688 PMC5895180

[B4] Bain RH Hurley MM (2011) A biogeographic synthesis of the amphibians and reptiles of Indochina. Bulletin of the American Museum of Natural History 360: 1–138. 10.1206/360.1

[B5] Baker RJ Bradley RD (2006) Speciation in mammals and the genetic species concept. Journal of Mammalogy 87(4): 643–662. 10.1644/06-MAMM-F-038R2.1 19890476 PMC2771874

[B6] Bannikova AA Zemlemerova ED Colangelo P Sözen M Sevindik M Kidov AA Dzuev RI Kryštufek B Lebedev VS (2015a) An underground burst of diversity–A new look at the phylogeny and taxonomy of the genus *Talpa* Linnaeus, 1758 (Mammalia, Talpidae) as revealed by nuclear and mitochondrial genes. Zoological Journal of the Linnean Society 175(4): 930–948. 10.1111/zoj.12298

[B7] Bannikova AA Zemlemerova ED Lebedev VS Aleksandrov DY Fang Y Sheftel BI (2015b) Phylogenetic position of the Gansu mole *Scapanulus oweni* Thomas, 1912 and the relationships between strictly fossorial tribes of the family Talpidae. Doklady Biological Sciences: Proceedings of the Academy of Sciences of the USSR, Biological Sciences Sections 464(1): 230–234. 10.1134/S0012496615050038 26530064

[B8] Barrow E MacLeod N (2008) Shape variation in the mole dentary (Talpidae, Mammalia). Zoological Journal of the Linnean Society 153(1): 187–211. 10.1111/j.1096-3642.2008.00376.x

[B9] Biltueva LS Vorobieva NV Lemskya NA Perelman PL Trifonov VA Panov VV Abramov AV Kawada S Serdukova NA Graphodatsky AS (2023) Chromosomal evolution of the Talpinae. Genes 14(7): 1472. 10.3390/genes14071472 37510376 PMC10379030

[B10] BirdLife International Vietnam Programme and FIPI (2001) Sourcebook of existing and proposed protected areas in Vietnam. BirdLife International Vietnam Programme and the Forest Inventory and Planning Institute, Hanoi. https://thiennhienviet.org.vn/sourcebook/source_book/index_EN.html

[B11] Bradley RD Baker RJ (2001) A test of the genetic species concept: Cytochrome-b sequences and mammals. Journal of Mammalogy 82(4): 960–973. 10.1644/1545-1542(2001)082<0960:ATOTGS>2.0.CO;2 PMC2771874 19890476

[B12] Bui TH (2022) Taxonomy study, distribution pattern, and phylogenetic relationship of shrew species (Mammalia, Soricomorpha) in Vietnam. PhD Thesis, Graduate University of Science and Technology, Hanoi, Vietnam. [In Vietnamese]

[B13] Bui TH Motokawa M Kawada S-I Abramov AV Nguyen TS (2020a) Skull variation in Asian moles of the genus *Euroscaptor* (Eulipotyphla, Talpidae) in Vietnam. Mammal Study 45(4): 265–280. 10.3106/ms2019-0058

[B14] Bui TH Motokawa M Ninh TH Le XC (2020b) A revision of the geographical distributions of the Southeast Asian shrews (*Crocidura dracula* and *C. fuliginosa*) based on new collection in Vietnam. Proceedings of the 4^th^ National Scientific Conference on Biological Research and Teaching in Vietnam, 3–10. 10.15625/vap.2020.0001

[B15] Burgin CJ Wilson DE Mittermeier RA Rylands AB Lacher TE Sechrest W (2020) Illustrated checklist of the mammals of the world. Vol. 2. Eulipotyphla to Carnivora. Lynx Edicions, Barcelona, 531 pp.

[B16] Center for Nature Conservation and Development (CEV) (2005) Sourcebook of existing and proposed protected areas in Vietnam. https://www.thiennhienviet.org.vn/sourcebook/source_book/North%20Central%20Coast/SB%20Pu%20Luong.htm

[B17] Dang NC Endo H Nguyen TS Oshida T Le XC Dang HP Lunde DP Kawada S-I Hayashida A Sasaki M (2008) Checklist of wild mammal species of Vietnam. Shoukado, Kyoto, 400 pp. [In English and Vietnamese]

[B18] Darwin C (1839) Journal of researches into the geology and natural history of the various countries visited by H.M.S. Beagle round the world. Henry Colburn Publishers, London, 615 pp. https://darwin-online.org.uk/content/frameset?viewtype=image&itemID=F11&pageseq=1

[B19] Darwin C (1859) On the origin of species by means of natural selection, or the preservation of favoured races in the struggle for life. John Murray Publishers, London, 502 pp. 10.5962/bhl.title.68064 PMC5184128 30164232

[B20] Edgar RC (2004) MUSCLE: Multiple sequence alignment with high accuracy and high throughput. Nucleic Acids Research 32(5): 1792–1797. 10.1093/nar/gkh340 15034147 PMC390337

[B21] Feijó A Wen Z Cheng J Ge D Xia L Yang Q (2019) Divergent selection along elevational gradients promotes genetic and phenotypic disparities among small mammal populations. Ecology and Evolution 9(12): 7080–7095. 10.1002/ece3.5273 31380035 PMC6662404

[B22] Hall TA (1999) BioEdit: A user-friendly biological sequence alignment editor and analysis program for Windows 95/98/NT. Nucleic Acids Symposium Series 41(2): 95–98. 10.14601/PHYTOPATHOL_MEDITERR-14998U1.29

[B23] Hammer Ø Harper DAT Ryan PD (2001) PAST: Paleontological statistics software package for education and data analysis. Palaeontologia Electronica 4(1).

[B24] He K Shinohara A Jiang XL Campbell KL (2014) Multilocus phylogeny of talpine moles (Talpini, Talpidae, Eulipotyphla) and its implications for systematics. Molecular Phylogenetics and Evolution 70: 513–521. 10.1016/j.ympev.2013.10.002 24140029

[B25] Herring SW (2007) Masticatory muscles and the skull: A comparative perspective. Archives of Oral Biology 52(4): 296–299. 10.1016/j.archoralbio.2006.09.010 17084804 PMC1853336

[B26] Hoang DT Chernomor O Haeseler AV Bui QM Le SV (2018) UFBoot2: Improving the ultrafast bootstrap approximation. Molecular Biology and Evolution 35(2): 518–522. 10.1093/molbev/msx281 29077904 PMC5850222

[B27] Hutterer R (2005) Order Soricomorpha. In: Wilson DE Reeder DM (Eds) Mammal species of the world.A taxonomic and geographic reference, 3^rd^ edn. The Johns Hopkins University Press, Baltimore, 220–311.

[B28] Huxley JS (1938) Species formation and geographical isolation. Proceedings of the Linnean Society of London 150(4): 253–264. 10.1111/j.1095-8312.1938.tb00182g.x

[B29] Kalyaanamoorthy S Minh BQ Wong TK Von Haeseler A Jermiin LS (2017) ModelFinder: Fast model selection for accurate phylogenetic estimates. Nature Methods 14(6): 587–589. 10.1038/nmeth.4285 28481363 PMC5453245

[B30] Kawada SI (2016) Morphological review of the Japanese mountain mole (Eulipotyphla, Talpidae) with the proposal of a new genus. Mammal Study 41(4): 191–205. 10.3106/041.041.0404

[B31] Kawada S Shinohara A Kobayashi S Harada M Oda S Lin L-K (2007) Revision of the mole genus *Mogera* (Mammalia: Lipotyphla: Talpidae) from Taiwan. Systematics and Biodiversity 5(2): 223–240. 10.1017/S1477200006002271

[B32] Kawada SI Yasuda M Shinohara A Lim BL (2008) Redescription of the Malaysian Mole as to be a true species, *Euroscaptor malayana* (Insectivora, Talpidae). Memoirs of the National Science Museum 45: 65–74.

[B33] Kawada S Nguyen TS Dang NC (2009) Moles (Insectivora, Talpidae, Talpinae) of Vietnam. Bulletin of the National Nature and Science Museum. Series A. Zoology: Analysis of Complex Systems, ZACS 35: 89–101.

[B34] Kawada SI Nguyen TS Dang NC (2012) A new species of mole of the genus *Euroscaptor* (Soricomorpha, Talpidae) from northern Vietnam. Journal of Mammalogy 93(3): 839–850. 10.1644/11-MAMM-A-296.1

[B35] Kocher TD Thomas WK Meyer A Edwards SV Pääbo S Villablanca FX Wilson AC (1989) Dynamics of mitochondrial DNA evolution in animals: Amplification and sequencing with conserved primers. Proceedings of the National Academy of Sciences of the United States of America 86(16): 6196–6200. 10.1073/pnas.86.16.6196 2762322 PMC297804

[B36] Kottler MJ (1978) Charles Darwin’s biological species concept and theory of geographic speciation: The transmutation notebooks. Annals of Science 35(3): 275–297. 10.1080/00033797800200251

[B37] Kryštufek B Motokawa M (2018) Family Talpidae (Moles, Desmans, Star–nosed moles and Shrew moles). In: Wilson DE Mittermeier RA (Eds) Handbook of the mammals of the world.Vol. 8. Insectivores, Sloths and Colugos. Lynx Editions, 552–619.

[B38] Le VK Nguyen XH Nguyen TN (2015) Zoogeography. Vietnam National University Press, Hanoi, 403 pp. [In Vietnamese]

[B39] Mayr E (1942) Systematics and the origin of species from the viewpoint of a zoologist. Columbia University Press, New York, 334 pp. https://ia801404.us.archive.org/13/items/in.ernet.dli.2015.20284/2015.20284.Systematics-And-The-Origin-Of-Species.pdf

[B40] Miller GS (1940) Notes on some moles from Southeastern Asia. Journal of Mammalogy 21(4): 442–444. 10.2307/1374883

[B41] Mindell DP Dick CW Baker RJ (1991) Phylogenetic relationships among megabats, microbats, and primates. Proceedings of the National Academy of Sciences of the United States of America 88(22): 10322–10326. 10.1073/pnas.88.22.10322 1658803 PMC52920

[B42] Motokawa M Lin L-K (2002) Geographic variation in the mole–shrew *Anourosorex squamipes*. Mammal Study 27(2): 113–120. 10.3106/mammalstudy.27.113

[B43] Nevo E (1985) Speciation in action and adaptation in subterranean mole rats: Patterns and theory. Bollettino di Zoologia 52(1–2): 65–95. 10.1080/11250008509440344

[B44] Nevo E (1999) Mosaic evolution of subterranean mammals: Regression, progression, and global convergence. Oxford University Press, Oxford, UK, 413 pp. 10.1093/oso/9780198575726.001.0001

[B45] Nguyen LT Schmidt HA von Haeseler A Bui QM (2015) IQ–TREE: A fast and effective stochastic algorithm for estimating maximum–likelihood phylogenies. Molecular Biology and Evolution 32(1): 268–274. 10.1093/molbev/msu300 25371430 PMC4271533

[B46] Okabe S Motokawa M (2024) Geographic variation of *Dymecodon pilirostris* (Eulipotyphla, Talpidae) with an insight into mountain island in Japan. Mammal Study 49(4): 345–357. 10.3106/ms2023-0013

[B47] Partha R Chauhan BK Ferreira Z Robinson JD Lathrop K Nischal KK Chikina M Clark NL (2017) Subterranean mammals show convergent regression in ocular genes and enhancers, along with adaptation to tunneling. eLife 6: e25884. 10.7554/eLife.25884 PMC5643096 29035697

[B48] Pérez MJ Barquez RM Díaz MM (2017) Morphology of the limbs in the semi-fossorial desert rodent species of *Tympanoctomys* (Octodontidae, Rodentia). ZooKeys 710: 77–96. 10.3897/zookeys.710.14033 PMC5674180 29118644

[B49] Pujolar JM Blom MPK Reeve AH Kennedy JD Marki PZ Korneliussen TS Freeman BG Sam K Linck E Haryoko T Iova B Koane B Maiah G Paul L Irestedt M Jønsson KA (2022) The formation of avian montane diversity across barriers and along elevational gradients. Nature Communications 13(1): 268. 10.1038/s41467-021-27858-5 PMC8755808 35022441

[B50] Ronquist F Teslenko M van der Mark P Ayres DL Darling A Höhna S Larget B Liu L Suchard MA Huelsenbeck JP (2012) MrBayes 3.2: Efficient Bayesian phylogenetic inference and model choice across a large model space. Systematic Biology 61(3): 539–542. 10.1093/sysbio/sys029 22357727 PMC3329765

[B51] Sansalone G Colangelo P Loy A Raia P Wroe S Piras P (2019) Impact of transition to a subterranean lifestyle on morphological disparity and integration in talpid moles (Mammalia, Talpidae). BMC Evolutionary Biology 19(1): 179. 10.1186/s12862-019-1506-0 31510915 PMC6739959

[B52] Sharp AC Dutel H Watson PJ Gröning F Crumpton N Fagan MJ Evans SE (2023) Assessment of the mechanical role of cranial sutures in the mammalian skull: Computational biomechanical modelling of the rat skull. Journal of Morphology 284(3): e21555. 10.1002/jmor.21555 PMC10107956 36630615

[B53] Shinohara A Kawada SI Nguyen TS Koshimoto C Endo H Dang NC Suzuki H (2014) Molecular phylogeny of East and Southeast Asian fossorial moles (Lipotyphla, Talpidae). Journal of Mammalogy 95(3): 455–466. 10.1644/13-MAMM-A-135

[B54] Shinohara A Kawada SI Nguyen TS Dang NC Sakamoto SH Koshimoto C (2015) Molecular phylogenetic relationships and intra–species diversities of three *Euroscaptor* spp. (Talpidae, Lipotyphla, Mammalia) from Vietnam. The Raffles Bulletin of Zoology 63: 366–375.

[B55] Sikes RS Gannon WL Animal Care and Use Committee of the American Society of Mammalogists (2011) Guidelines of the American Society of Mammalogists for the use of wild mammals in research. Journal of Mammalogy 92(1): 235–253. 10.1644/10-MAMM-F-355.1 PMC5909806 29692469

[B56] Smith AL Grosse IR (2016) The biomechanics of zygomatic arch shape. The Anatomical Record: Advances in Integrative Anatomy and Evolutionary Biology 299(12): 1734–1752. 10.1002/ar.23484 27870343 PMC5726875

[B57] Smith AT Xie Y (Eds.) (2008) A Guide to the Mammals of China. Princeton University Press, Princeton, New Jersey, 544 pp.

[B58] Sterling EJ Hurley MM (2005) Conserving biodiversity in Vietnam: applying biogeography to conservation research. Proceedings of the California Academy of Sciences 56(Suppl. I, 9): 98–114.

[B59] Sterling EJ Hurley MM Le DM (2006) Vietnam: a natural history. Yale University Press, New Haven & London, [xviii +] 423 pp. 10.12987/9780300128215

[B60] Tamura K Stecher G Kumar S (2021) MEGA11: Molecular evolutionary genetics analysis version 11. Molecular Biology and Evolution 38(7): 3022–3027. 10.1093/molbev/msab120 33892491 PMC8233496

[B61] Tanabe AS (2011) Kakusan 4 and Aminosan: Two programs for comparing nonpartitioned, proportional and separate models for combined molecular phylogenetic analyses of multilocus sequence data. Molecular Ecology Resources 11(5): 914–921. 10.1111/j.1755-0998.2011.03021.x 21592310

[B62] Tordoff AW Tran QB Nguyen TD Le MH (2004) Source Book of Existing and Proposed Protected Areas in Vietnam. Vol. 1, Second Edition. BirdLife International Indochina, Hanoi. https://thiennhienviet.org.vn/

[B63] Tordoff AW Baltzer MC Fellowes JR Pilgrim JD Langhammer PF (2012) Key biodiversity areas in the Indo–Burma hotspot: Process, progress and future directions. Journal of Threatened Taxa 4(8): 2779–2787. 10.11609/JoTT.o3000.2779-87

[B64] Vaidya G Lohman DJ Meier R (2011) SequenceMatrix: Concatenation software for the fast assembly of multi–gene datasets with character set and codon information. Cladistics: The International Journal of the Willi Hennig Society 27(2): 171–180. 10.1111/j.1096-0031.2010.00329.x 34875773

[B65] Zemlemerova ED Bannikova AA Abramov AV Lebedev VS Rozhnov VV (2013) New data on molecular phylogeny of the East Asian moles. Doklady Biological Sciences: Proceedings of the Academy of Sciences of the USSR, Biological Sciences Sections 451(1): 257–260. 10.1134/S0012496613040200 23975471

[B66] Zemlemerova ED Bannikova AA Lebedev VS Rozhnov VV Abramov AV (2016) Secrets of the underground Vietnam: An underestimated species diversity of the Asian moles (Lipotyphla, Talpidae, *Euroscaptor*). Trudy Zoologicheskogo Instituta 320(2): 193–220. 10.31610/trudyzin/2016.320.2.193

