# Supplementary material for: ﻿Euroscaptor darwini sp. nov., a new species of mole (Mammalia, Eulipotyphla, Talpidae) from the north-central mountains in Vietnam
Source: Zookeys. 2025 Oct 10;1255:239–74. doi: 10.3897/zookeys.1255.161942 (PMC12534793; doi:10.3897/zookeys.1255.161942)
Supplement: Supplementary material 1 — Morphological [file zookeys-1255-239_article-161942__-s001.docx]

**Supplementary material 1.** List of craniodental measurements used in this study.

| **No.** | **Character** | **Abbreviations** |
| --- | --- | --- |
| **Cranium** | | |
| **1** | **GLS** | Greatest length of skull (from the front of the 1st upper incisor to the most projecting point of the occipital region). |
| **2** | **BB** | Breadth of braincase at the posterior roots of zygomatic arches. |
| **3** | **IOB** | Greatest interorbital breadth (least breadth of the interorbital constriction). |
| **4** | **BIOF** | Breadth between infraorbital foramina |
| **5** | **RB** | Rostral breadth at the canines |
| **6** | **LZA** | Inner length of zygomatic arch |
| **7** | **PZRW** | Posterior roots of zygomatic arches breadth |
| **8** | **ACRB** | Anterior condylo root breadth |
| **9** | **FMW** | Foramen magnum Width |
| **10** | **CCoL** | Canine–condylo length (distance from the exoccipital condyle to the most anterior part of the canine). |
| **11** | **PL** | Palatal length (distance from the anterior tip of 1st incisor to the posterior lip of the palate) |
| **12** | **I^1^–M^3^** | Maxillary toothrow length (distance from the front of upper canine to the back of the crown of the 3rd molar) |
| **13** | **P^1^–P^4^** | Upper premolar crown length (from the front of the 1st upper premolar to the last premolar) |
| **14** | **M^1^–M^3^** | Upper molar crown length (from the front of the 1st upper molar to the last molar). |
| **15** | **M^2^–M^2^W** | Width across 2nd upper molars (greatest width across the outer borders of 2nd upper molars) |
| **16** | **CCW** | Width across the upper canines (greatest width across the outer borders of the upper canines). |
| **17** | **PWCC** | Anterior palatal width (least distance between the inner borders of the upper canines) |
| **18** | **PWM^2^M^2^** | Inner width across 2nd upper molars (distance between inner borders of 2nd upper molars) |
| **19** | **PWM^3^M^3^** | Posterior palatal width (least distance between the inner borders of the last upper molars) |
| **20** | **C–M^3^** | Upper canine–molar length (distance from the front of upper canine to the back of the crown of the third molar). |
| **21** | **C–P^4^** | Upper canine–premolar length (distance from the front of the upper canine to the back of the crown of the last premolar). |
| **22** | **I1–P4** | Upper incisor–last premolar length (from the front of the upper incisor to the back of the crown of the last premolar) |
| **23** | **I^1^–P^3^** | Upper incisor–3rd premolar length (from the front of the upper incisor to the back of the crown of the third premolar) |
| **24** | **I^1^–C** | Upper incisor–canine (from the front of the upper incisor to the back of the crown of the canine) |
| **25** | **P^4^–M^3^** | Upper molariform toothrow length (from the posterior upper premolar to the last molar). |
| **Mandible** | | |
| **26** | **ML** | Mandible length (distance from the anterior rim of the alveolus of the first lower incisor to the most posterior part of the condyle). |
| **27** | **CAL** | Coronoid process–angular process length (distance from the tip of the coronoid process to the tip of the angular process) |
| **28** | **MH** | Mandible height at the coronoid process (distance from the tip of the coronoid process to the apex of the indentation on the inferior surface of the ramus adjacent to the angular process) |
| **29** | **i_1_–m_3_** | Mandibular toothrow length (distance from the front of 1st lower incisor to the back of the crown of the 3nd molar) |
| **30** | **i_1_–p_4_** | Lower incisor–last premolar length (from the front of the lower incisor to the back of the crown of the last premolar) |
| **31** | **p_1_–m_3_** | Lower 1st premolar–molar length (from the front of the 1st lower premolar to the back of the crown of the last molar) |
| **32** | **p_4_–m_3_** | Lower molariform toothrow length (from the posterior lower premolar to the last molar) |
| **33** | **m_1_–m_3_** | Lower molar crown length (from the front of the 1st lower molar to the last molar) |
| **34** | **i_1_–c** | Lower incisor-canine length (from the front of the 1st lower incisor to canine) |
| **35** | **i_1_–p_1_** | Lower 1st incisor–1st premolar length (from the front of the 1st lower incisor to the back of the crown of the 1st premolar) |
| **36** | **p_1_–p_4_** | Lower premolar crown length (from the front of the 1st lower premolar to the last premolar) |
