# Supplementary material for: ﻿Euroscaptor darwini sp. nov., a new species of mole (Mammalia, Eulipotyphla, Talpidae) from the north-central mountains in Vietnam
Source: Zookeys. 2025 Oct 10;1255:239–74. doi: 10.3897/zookeys.1255.161942 (PMC12534793; doi:10.3897/zookeys.1255.161942)
Supplement: Supplementary material 2 — List of skull specimens, morphological [file zookeys-1255-239_article-161942__-s002.docx]

**Supplementary material 2.** List of *Euroscaptor* skull specimens from Vietnam examined in this study.

| **No.** | **Locality** | **Species** | **Voucher** |
| --- | --- | --- | --- |
| **1** | Pu Luong, Nghe An | *E*. *darwini* sp. nov. | NTS 2024.PL.01 |
| **2** | Pu Luong, Nghe An |  | NTS.2025.PL.02 |
| **3** | Pu Luong, Nghe An |  | NTS.2025.PL.03 |
| **4** | Pu Luong, Nghe An |  | NTS.2025.PL.04 |
| **5** | Pu Luong, Nghe An |  | NTS.2025.PL.05 |
| **6** | Cuc Phuong, Ninh Binh | *E*. *subanura* | NTS 2017.08–01 |
| **7** | Na Hang, Tuyen Quang |  | SIK 0888 |
| **8** | Na Hang, Tuyen Quang |  | SIK 0889 |
| **9** | Tam Dao, Tuyen Quang |  | SIK 0882 |
| **10** | Tam Dao, Tuyen Quang |  | SIK 0883 |
| **11** | Tam Dao, Tuyen Quang |  | SIK 0884 |
| **12** | Tam Dao, Tuyen Quang |  | SIK 0875 |
| **13** | Tam Dao, Tuyen Quang |  | SIK 0876 |
| **14** | Pu Huong, Nghe An |  | SIK 0890 |
| **15** | Xuan Son, Phu Tho |  | SIK 0931 |
| **16** | Xuan Son, Phu Tho |  | SIK 0932 |
| **17** | Xuan Son, Phu Tho |  | SIK 0933 |
| **18** | Xuan Son, Phu Tho |  | SIK 0934 |
| **19** | Xuan Son, Phu Tho |  | SIK 0935 |
| **20** | Xuan Son, Phu Tho |  | SIK 0936 |
| **21** | Xuan Son, Phu Tho |  | SIK 0937 |
| **22** | Xuan Son, Phu Tho |  | SIK 0938 |
| **23** | Xuan Son, Phu Tho |  | SIK 0939 |
| **24** | Xuan Nha, Son La |  | XN–16 |
| **25** | Xuan Nha, Son La |  | XN–58 |
| **26** | Xuan Nha, Son La |  | XN–20 |
| **27** | Ngoc Linh, Kon Tum | *E*. *ngoclinhensis* | Motokawa–641 |
| **28** | Ngoc Linh, Kon Tum |  | Motokawa–637 |
| **29** | Ngoc Linh, Kon Tum |  | Motokawa–642 |
| **30** | Ngoc Linh, Kon Tum |  | NTS 2019.NL.11 |
| **39** | Hon Ba, Khanh Hoa | *E*. *parvidens* | Motokawa–0421 |
| **40** | Hon Ba, Khanh Hoa |  | Motokawa–0427 |
| **41** | Hon Ba, Khanh Hoa |  | Motokawa–428 |
| **42** | Hon Ba, Khanh Hoa |  | Motokawa–0432 |
| **43** | Hon Ba, Khanh Hoa |  | Motokawa–0433 |
| **44** | Hon Ba, Khanh Hoa |  | Motokawa–435 |
| **45** | Hon Ba, Khanh Hoa |  | Motokawa–0434 |
| **46** | Hon Ba, Khanh Hoa |  | Motokawa–0436 |
| **47** | Hon Ba, Khanh Hoa |  | Motokawa–0437 |
| **48** | Hon Ba, Khanh Hoa |  | Motokawa–0439 |
| **49** | Nam Nung, Dak Nong |  | N.NU.2017.09 |
| **50** | Nam Nung, Dak Nong |  | N.NU.2017.05 |
| **51** | Nam Nung, Dak Nong |  | N.NU.2017.02 |
| **52** | Tam Dao, Vinh Phuc | *E*. *kuznetsovi* | SIK–774 |
| **53** | Tam Dao, Vinh Phuc |  | SIK–777 |
| **54** | Tam Dao, Vinh Phuc |  | SIK–788 |
| **55** | Nguyen Binh, Cao Bang |  | SIK–866 |
| **56** | Nguyen Binh, Cao Bang |  | CB 16 |
| **57** | Tam Dao, Vinh Phuc |  | SIK–775 |
| **58** | Tam Dao, Vinh Phuc |  | SIK–776 |
| **59** | Tam Dao, Vinh Phuc |  | SIK–778 |
| **60** | Tam Dao, Vinh Phuc |  | SIK–779 |
| **61** | Tam Dao, Vinh Phuc |  | SIK–781 |
| **62** | Nguyen Binh, Cao Bang |  | SIK–867 |
| **63** | Hoang Lien, Lao Cai | *E*. *orlovi* | Motokawa–0846 |
| **64** | Sa Pa, Lao Cai |  | SIK–981 |
| **65** | Sa Pa, Lao Cai |  | SIK–982 |
