# Supplementary material for: ﻿Euroscaptor darwini sp. nov., a new species of mole (Mammalia, Eulipotyphla, Talpidae) from the north-central mountains in Vietnam
Source: Zookeys. 2025 Oct 10;1255:239–74. doi: 10.3897/zookeys.1255.161942 (PMC12534793; doi:10.3897/zookeys.1255.161942)
Supplement: Supplementary material 3 — List of Cyt b sequences, phylogenetic [file zookeys-1255-239_article-161942__-s003.docx]

**Supplementary material 3.** The list of Cyt *b* sequences used for pairwise comparisons.

| **Species** | **n** | **Collecting Locality** | **Elevation (m)** | **Voucher** | **DNA Accession Number** | **Reference** |
| --- | --- | --- | --- | --- | --- | --- |
|  |  |  |  |  | **Cyt *b* (1140bp)** |  |
| *Eurocaptor darwini* sp. nov. | 5 | Pu Luong Nature Reserve, Thanh Hoa | 935–950 m | [NTS.2024.PL](http://nts.2024.pl/).01 | PV745521 | *This study* |
|  |  |  |  | NTS.2025.PL.02 | PV745522 |  |
|  |  |  |  | NTS.2025.PL.03 | PV745523 |  |
|  |  |  | 1090–1125 m | NTS.2025.PL.04 | PV745524 |  |
|  |  |  |  | NTS.2025.PL.05 | PV745525 |  |
| *E*. *subanura* | 30 | Tam Dao, Vinh Phuc | 250–320 m | SIK0882, SIK0883 | LC013281, LC013282 | *Shinohara et al*. *2015* |
|  |  |  |  | SIK0875, SIK0876 | LC013279, LC013280 |  |
|  |  |  |  | ZIN101534 | KX164261 |  |
|  |  | Na Hang, Tuyen Quang | — | SIK0913–SIK0919 | LC013283–LC013289 |  |
|  |  |  | — | SIK0922, SIK0924, SIK0930, SIK0932, SIK0936–SIK0939 | LC013290–LC013297 |  |
|  |  | Xuan Son, Phu Tho | 300–420 m | ZIN 101924–ZIN 101927 | KX164264–KX164266, KX164268 | *Zemlemerova et al*. *2016* |
|  |  | — | — | ZIN 102248 | KX164267 |  |
|  |  | — | — | AVA 14–117 | KX164269 |  |
|  |  | — | — | AAV 12–276 | KX164260 |  |
|  |  | Ba Vi, Ha Noi | 320–570 m | ZIN 101897, ZIN 101898 | KX164262, KX164263 | *Bannikova et al*. *2015* |
|  |  |  |  | ZIN 101900 | KP995370 |  |
| *E*. *parvidens* | 13 | Bi Dup–Nui Ba Nature Reserve, Lam Dong | 1400 m | ZIN98916, ZIN98918 | KC481338, KC481340 | *Zemlemerova et al*. *2013* |
|  |  | Bao Lam, Lam Dong | 790–830 m | ZIN 101901, ZIN 101902 | KX164254, KX164256 | *Zemlemerova et al*. *2016* |
|  |  |  |  | ZMMU S–195070, ZMMU S–195971 | KX164255, KX164257 |  |
|  |  | Chu Yang Sin National Park, Dak Lak | 900–1200 m | ZIN 101920–ZIN 101923 | KX164249–KX164252 |  |
|  |  | Nam Nung, Dak Nong | 1000 m | N.NU.2017.02 | BM6 | *Bui 2022* |
| *E*. *ngoclinhensis* | 2 | Song Hinh, Phu Yen | — | AAV.20140, AAV.20133 | FV2, FV7 | *This study* |
| *E*. *kuznetsovi* | 6 | Tam Dao National Park, Vinh Phuc | 700–1300 m | SIK0775–SIK0777 | AB823108–AB823110 | *Shinohara et al*. *2014* |
|  |  | Nguyen Binh, Cao Bang | 800–1200 m | SIK0865–SIK0867 | AB823114–AB823116 |  |
| *E*. *orlovi* | 3 | Sapa, Hoang Lien National Park, Lao Cai | 1900–2330 m | SIK0820–SIK0822 | AB823111–AB823113 |  |
| *E*. *klossi* | 2 | Mae Sa Long, Chiang Rai, Thailand | — | SIK0673, SIK0674 | AB823106, AB823107 |  |
| *E*. *malayana* | 2 | Cameron Highlands, Phang, Malaysia | — | SIK0550, SIK0557 | AB185151, AB185152 | *Shinohara et al*. *2004* |
