# Supplementary material for: ﻿Euroscaptor darwini sp. nov., a new species of mole (Mammalia, Eulipotyphla, Talpidae) from the north-central mountains in Vietnam
Source: Zookeys. 2025 Oct 10;1255:239–74. doi: 10.3897/zookeys.1255.161942 (PMC12534793; doi:10.3897/zookeys.1255.161942)
Supplement: Supplementary material 5 — List of DNA accession number, phylogenetic, genomic [file zookeys-1255-239_article-161942__-s005.docx]

**Supplementary material 5.** *Euroscaptor* specimens with DNA Accession Number used in the Phylogenetic analyses based on concatenated mitochondrial Cyt *b* and 12S rRNA gene sequences. Asterisks indicate holotype (*) and hash sign indicate paratype (#) specimens of both *E*. *darwini* and *E*. *subanura*.

| **Species** | **n** | **Collecting Locality** | **Voucher** | **Sex** | **DNA Accession Number** | | **Reference** |
| --- | --- | --- | --- | --- | --- | --- | --- |
|  |  |  |  |  | **Cyt *b* (1140bp)** | **12S (846bp)** |  |
| *E*. *darwini* sp. nov. | 4 | Pu Luong Nature Reserve, Thanh Hoa | [NTS.2024.PL](http://nts.2024.pl/).01 | ♂* | PV745521 | PV740727 | *This study* |
|  |  |  | NTS.2025.PL.02 | ♀# | PV745522 | PV740728 |  |
|  |  |  | NTS.2025.PL.03 | ♀# | PV745523 | PV740729 |  |
|  |  |  | NTS.2025.PL.05 | ♀# | PV745525 | PV740730 |  |
| *E*. *subanura* | 19 | Tam Dao, Tuyen Quang | SIK0882 | ♂* | LC013281 | LC013300 | *Shinohara et al*. *2015* |
|  |  |  | SIK0883 | ♀# | LC013282 | LC013301 |  |
|  |  |  | SIK0875 | ♂# | LC013279 | LC013298 |  |
|  |  |  | SIK0876 | ♂# | LC013280 | LC013299 |  |
|  |  | Na Hang, Tuyen Quang | SIK0913 | ♂ | LC013283 | LC013302 |  |
|  |  |  | SIK0914 | ♂ | LC013284 | LC013303 |  |
|  |  |  | SIK0915 | ♀ | LC013285 | LC013304 |  |
|  |  |  | SIK0916 | ♀ | LC013286 | LC013305 |  |
|  |  |  | SIK0917 | ♂ | LC013287 | LC013306 |  |
|  |  |  | SIK0918 | ♂ | LC013288 | LC013307 |  |
|  |  |  | SIK0919 | ♂ | LC013289 | LC013308 |  |
|  |  |  | SIK0922 | ♂ | LC013290 | LC013309 |  |
|  |  |  | SIK0924 | ♂ | LC013291 | LC013310 |  |
|  |  |  | SIK0930 | ♂ | LC013292 | LC013311 |  |
|  |  |  | SIK0932 | ♀ | LC013293 | LC013312 |  |
|  |  |  | SIK0936 | ♀ | LC013294 | LC013313 |  |
|  |  |  | SIK0937 | ♀ | LC013295 | LC013314 |  |
|  |  |  | SIK0938 | ♀ | LC013296 | LC013315 |  |
|  |  |  | SIK0939 | ♀ | LC013297 | LC013316 |  |
| *E*. *ngoclinhensis* | 2 | Song Hinh, Phu Yen | AAV.20140 | — | FV2 | 4FV2 | *This study* |
|  |  |  | AAV.20133 | — | FV7 | 4FV7 |  |
| *E*. *parvidens* | 1 | Nam Nung, Dak Nong | N.NU.2017.02 | — | BM6 | 4BM6 | *Bui 2022* |
| *E*. *kuznetsovi* | 6 | Tam Dao, Vinh Phuc | SIK0775 | — | AB823108 | AB823143 | *Shinohara et al*. *2014* |
|  |  |  | SIK0776 | — | AB823109 | AB823144 |  |
|  |  |  | SIK0777 | — | AB823110 | AB823145 |  |
|  |  | Nguyen Binh, Cao Bang | SIK0865 | — | AB823114 | AB823149 |  |
|  |  |  | SIK0866 | — | AB823115 | AB823150 |  |
|  |  |  | SIK0867 | — | AB823116 | AB823151 |  |
| *E*. *orlovi* | 3 | Sapa, Hoang Lien National Park, Lao Cai | SIK0820 | — | AB823111 | AB823146 |  |
|  |  |  | SIK0821 | — | AB823112 | AB823147 |  |
|  |  |  | SIK0822 | — | AB823113 | AB823148 |  |
| *E*. *klossi* | 2 | Mae Sa Long, Chiang Rai, Thailand | SIK0673 | — | AB823106 | AB823141 |  |
|  |  |  | SIK0674 | — | AB823107 | AB823142 |  |
| *E*. *malayana* | 2 | Cameron Highlands, Pahang, Malaysia | SIK0550 | — | AB185151 | AB185153 | *Shinohara et al*. *2004* |
|  |  |  | SIK0557 | — | AB185152 | AB185154 |  |
